# A Wearable Healthcare Platform Integrated with Biomimetical Ions Conducted Metal–Organic Framework Composites for Gas and Strain Sensing in Non‐Overlapping Mode

**DOI:** 10.1002/advs.202207663

**Published:** 2023-04-20

**Authors:** Qingqing Zhou, Zixun Geng, Long Yang, Bo Shen, Zitong Kan, Yu Qi, Songtao Hu, Biao Dong, Xue Bai, Lin Xu, Hongwei Song, Luquan Ren

**Affiliations:** ^1^ State Key Laboratory of Integrated Optoelectronics College of Electronic Science and Engineering Jilin University Changchun 130012 P. R. China; ^2^ Key Laboratory of Bionic Engineering Ministry of Education College of Biological and Agricultural Engineering Jilin University Changchun 130025 P. R. China

**Keywords:** DMA/strain sensors, flexible wearable electronics, health‐monitoring, ionically artificial synapse

## Abstract

Intelligent wearable devices are essential for telemedicine healthcare as they enable real‐time monitoring of physiological information. Elaborately constructing synapse‐inspired materials provides a crucial guidance for designing high‐performance sensors toward multiplex stimuli response. However, a realistic mimesis both in the “structure and sense” of biological synapses to obtain advanced multi‐functions is still challenging but essential for simplifying subsequent circuit and logic programs. Herein, an ionic artificial synapse integrated with Ti_3_CNT*_x_* nanosheets in situ grown with zeolitic imidazolate framework flowers (ZIF‐L@Ti_3_CNT*_x_* composite) is constructed to concurrently mimic the structure and working mechanism of the synapse. The flexible sensor of the bio‐inspired ZIF‐L@Ti_3_CNT*_x_* composite exhibits excellent dual‐mode dimethylamine (DMA) and strain‐sensitive response with non‐overlapping resistance variations. The specific ions conduction working principle triggered by DMA gas or strain with the assistance of humidity is confirmed by the density functional theory simulation. Last, an intelligent wearable system is self‐developed by integrating the dual‐mode sensor into flexible printed circuits. This device is successfully applied in pluralistic monitoring of abnormal physiological signals of Parkinson's sufferers, including real‐time and accurate assessment of simulated DMA expiration and kinematic tremor signals. This work provides a feasible routine to develop intelligent multifunctional devices for upsurging telemedicine diagnosis.

## Graphical abstract

An ionic artificial synapse device that couples ions conducted ZIF‐L and the Ti_3_CNT*_x_* is designed by a realistic mimesis both in the “structure and sense” of synapses. It shows excellent dual‐mode non‐overlapping perception of dimethylamine gas and strain. Last, an intelligently wearable platform is self‐developed by integrating the device into flexible circuits to real‐time monitor multiple Parkinson‐related physiological signals.

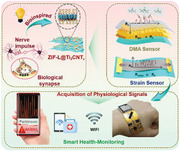

## 1 Introduction

Development of multifunctional, flexible, and smart electronic devices has revolutionized the telemedicine industry.^[^ ^1^ ^]^ Wearable healthcare devices for diagnosis of body temperature, pulse, heart rate, respiratory rate, expiratory markers, and other physiological information have been attracting extensive attention in non‐invasive health and telemedical monitoring. Compared with traditional rigid counterparts, wearable devices possess numerous merits, that is, light weight, portability, flexibility to withstand mechanical deformation, high integration, and on‐skin conformability, meeting the needs of full‐scale human motion detection, which is not possible with traditional rigid electronic devices. Moreover, carefully designed functional materials with 3D, pore, Janus, and bionic structures can significantly improve the sensitivity and extend the working window of wearable sensors, guaranteeing excellent sensing performances of the device on the deformed substrate. These unique characteristics enable the wearable devices to show response to multiple variables, such as gas, strain, stress, temperature signals, and even make the wearable devices exhibit comparable or better response values to those of rigid devices. Intelligent wearable sensing equipment can assist individuals in capturing the early‐warning signals of chronic diseases such as Parkinson's disease (PD), Amyotrophic lateral sclerosis and others,^[^ ^2^ ^]^ as well as distinguish the abnormal physiological behavior in the telemedicine diagnosis. In fact, weak changes in biological signals produced in the early stages of the disease are not easily perceived, and screening for single biomarkers is not sufficiently reliable for disease diagnosis. Therefore, it is essential to identify multiple weak physiological signals related to lesions over time synchronously and multidimensionally to promote practical processes. Until now, most multifunctional wearable devices for diagnosis are integrated discrete units, which require coordination with intricate circuit and complex logic programming; thus, suffering from cross‐interference in signal transmission.^[^ ^3^ ^]^ Another overlooked point is that wearable sensors should possess good universality on various scaffolding substrates to support versatile applications in more scenarios.^[^ ^4^ ^]^ Accordingly, developing single and intelligent sensors with multiple functions to simplify the human–computer interaction mode and make wearable devices more efficient and universal is significant. However, as the core sensing component, the construction of effective sensing materials with adjustable and diacritical response characteristics to meet the above requirements remains a great challenge.

Synapses are the most efficient and sensitive perceptual units in the body, processing information through distributed, fault‐tolerant, and event‐driven computing.^[^ ^5^ ^]^ They have the merits of a compact structure, high efficiency, and parallel reliability.^[^ ^6^ ^]^ Mimicking synapses as the building blocks of neural processing is one of the promising alternatives to construct multifunctional sensors that can realize accurate recognition of multiple signals. Learning from the structure of synapses, Chen et al. designed an artificial motion sensory system that integrated a tribo‐nanogenerator and a synaptic transistor array to achieve multimodal rotation and visual recognition functions with 89.82% accuracy.^[^ ^7^ ^]^ Bao et al. developed a monosynaptic reflex arc by coupling ring oscillators with artificial synaptic transistors for collecting pressure stimuli (1–80 kilopascals), distinguishing Braille characters, and actuating muscle motor.^[^ ^5a^ ^]^ These studies demonstrate the great potential of biomimetic electronics with synaptic features in signal transduction and perceptual interaction. In fact, ion transduction plays an indispensable role during the working process of nerve synapses. ^[^ ^8^ ^]^ In this process, multiple action potentials of postsynaptic membrane are triggered by the specific ion transmission when released neurotransmitters are captured by biological receptors via synapse.^[^ ^6b^ ^]^ However, the currently reported bionic synaptic device is mostly confined to the electrical signal modulation (i.e., the resistance, capacitance, or voltage change) to realize the mechanoelectrical transduction, which is different from the real neural response based on ion‐mediated signal transduction mechanisms in the human body. Beyond biomimetic in configuration, simultaneously endowing the artificial synapses with similar working mechanism can more authentically simulate and realize the advanced features of synapses. A realistic mimesis both in the “structure and sense” of biological synapses would be very conducive to integrating multiple sensory feedback and real‐time responses to external stimuli.

Zeolitic imidazolate frameworks (ZIFs), as a subbranch of the metal–organic frameworks (MOFs) family, have been extensively exploited in various fields due to prominent specific surface area, customized pore structures, and excellent biocompatibility.^[^ ^9^ ^]^ In particular, the network composed of abundant topological structure and free imidazole‐ligand units provides storage sites and fast diffusion channels for hydrogen protons.^[^ ^10^ ^]^ An intriguing ion shuttle feature enlightens a new spark of constructing ZIF‐based artificial synaptic receptors to imitate the information conveyance of the synaptic cleft. However, ZIFs generally show poor intrinsic conductivity owing to the insulative organic ligands, which cannot afford the fast transmission of hydrogen protons between ZIFs. As emerging 2D transition metal carbides and carbonitrides, MXene could be a potential supplement to achieve the effective transmission of biological neural signals.^[^ ^11^ ^]^ Sufficient electrochemical active sites, excellent electrical conductivity, and adjustable layer spacing of 2D MXene all provide powerful assistance for accelerating the proton migration.^[^ ^12^ ^]^ More importantly, the protons transition triggered in the ZIF receptors may directly couple with the 2D MXene surfaces with rich hydroxyl terminal and are expected to fast and effectively sense the external stimuli (gas or strain) into electrical nerve‐like signals.

Inspired by the biological neuromorphic sensing system, an ionic artificial synapse is established to timely and diacritically detect the multiple human physiological signals in this work. Combining the specific proton conduction ability and flower‐like structure, ZIF‐L particles are first in situ grown on the Ti_3_CNT*_x_* nanosheets (ZIF‐L@Ti_3_CNT*_x_* composite) to concurrently mimic the structure and working mechanism of synapse and postsynaptic membrane, respectively. The developed multisensory device based on bio‐spired ZIF‐L@Ti_3_CNT*_x_* composite achieves excellent ion diffusion dynamics, various substrate compatibilities, and importantly, the non‐overlapping gas and strain sensing characteristics. In addition, through the introduction of an ion conduction mechanism, the dual‐mode sensor also effectively utilizes the high humidity environment of human (such as exhaled breath and sweating) to accelerate the ions conduction capacity, that has an important impact on the sensor performance. Furthermore, a bio‐inspired intelligent sensing system is developed by integrating the multifunctional sensor with a flexible circuit to monitor Parkinson‐related physiological metrics, including abnormal expiratory markers and somatic kinematic dysfunctional tremors. This work offers a broad prospect for bio‐inspired signal processing and transduction in integrated telemedicine diagnosis and human–computer intelligence interaction systems.

## 2 Results and Discussion

### 2.1 Preparation and Characterization of Bio‐Inspired ZIF‐L@Ti_3_CNT*_x_* Composites

**Figure 1:**
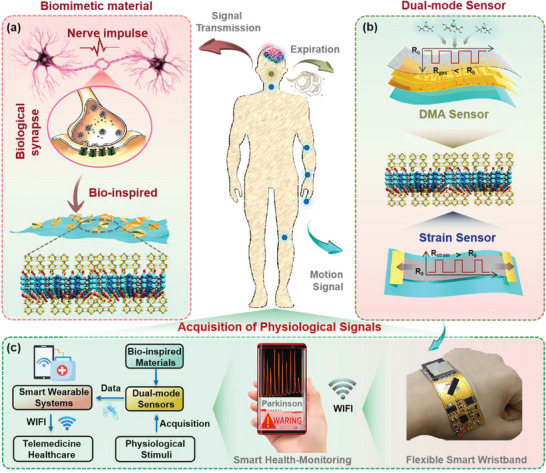
Concept and design of this study. a) The design of ZIF‐L@Ti_3_CNT*_x_* composites inspired by the synaptic structure. b) Construction of the flexible dual‐mode gas and strain sensors by applying the bio‐inspired ZIF‐L@Ti_3_CNT*_x_* composites. c) A smart wearable system integrated flexible circuit for point‐of‐care health‐monitoring of Parkinson's disease.

Ingenious synaptic structures and unique ion conduction functions in the olfactory and tactile organs ensure powerful signal processing capabilities.^[^ ^8^ ^]^ Benefiting from the advantages of synaptic and unidirectional transmission, information identification can proceed efficiently and rapidly. Inspired by the structure and function of biological synapses, a flexible intelligent wearable system integrated with the bioinspired ZIF‐L@Ti_3_CNT*_x_* materials for the dual‐mode perception of dimethylamine (DMA)and strain is constructed (**Figure** **1**). In the micro‐nano structure of ZIF‐L@Ti_3_CNT*_x_* composites, ZIF‐L particles and MXene nanosheets are adopted to imitate the receptors and postsynaptic membrane, respectively, which are tightly coupled by in situ growth of the flower‐like ZIF‐L particles onto the MXene nanosheets (Figure 1a). Herein, the ZIF‐L particles specifically capture external stimuli (gas/tremor) to trigger internal ion conduction, and further combine hydrogen bond channels on the surfaces of MXene nanosheets to promote efficient ion transport. The bio‐inspired composites are then used as active sensing layers to construct flexible gas and strain sensors for the simultaneous acquisition of physiological signals of patients with Parkinson's disease, such as expiratory DMA gas and body movements determination (Figure 1b). Last, the fabricated dual‐mode sensors are integrated with a flexible circuit for wireless, real‐time, and remote monitoring of physiological signals for point‐of‐care applications (Figure 1c).

**Figure 2:**
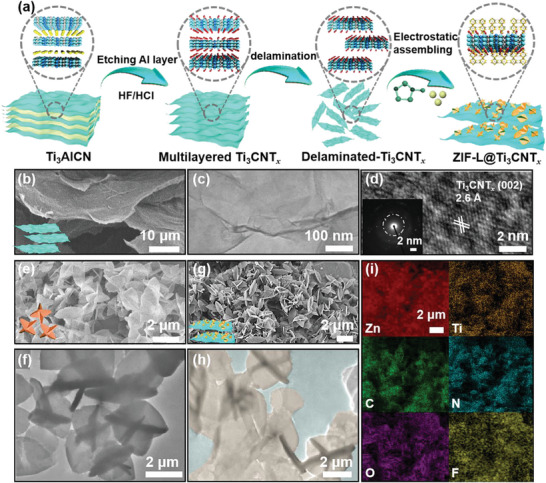
a) Schematic illustration of the synthetic process of the Ti_3_CNT*_x_* nanosheets and ZIF‐L@Ti_3_CNT*_x_* composite. b) SEM, c) TEM, and d) HRTEM images of the delaminated Ti_3_CNT*_x_* nanosheets (inset, SAED pattern). e) SEM and f) TEM images of the ZIF‐L particles, respectively. g) SEM, h) TEM (the light yellow and green fillings indicate the ZIF‐L particles and Ti_3_CNT*_x_* nanosheets, respectively), and i) EDX mapping images of the ZIF‐L@Ti_3_CNT*_x_* composite.

The fabrication procedure of the bio‐inspired hierarchical ZIF‐L@Ti_3_CNT*_x_* composites is schematically displayed in **Figure** **2**a. First, the parental bulk Ti_3_AlCN is selectively chemically etched by using a mixture of LiF and HCl acid, resulting in a multilayered and close‐packed Ti_3_CNT*_x_* structure, as depicted in its cross‐sectional scanning electron microscope (SEM) morphology (Figure S1, Supporting Information). After the intercalation and exfoliation of multilayer Ti_3_CNT*_x_*, mono or few‐layered nanosheets with smooth planar surface and large sizes are obtained (Figure 2b,c). The thickness of Ti_3_CNT*_x_* nanosheets recorded by atomic‐force microscopy is proven to be in the range of 3.76 ± 0.8 nm (Figure S2, Supporting Information), corresponding to the thickness of bi‐layered Ti_3_CNT*_x_* nanosheets. A clear lattice fringe related to the (002) plane of Ti_3_CNT*_x_* in the high‐resolution transmission electron microscopy (HRTEM) images and selected‐area electron diffraction patterns (SAED) is distinctly observed, indicating a high crystallinity quality (Figure 2d).^[^ ^13^ ^]^ The SEM and TEM images of pristine ZIF‐L particles are shown in Figure 2e,f, in which the flower‐like particles with sizes ranging from 2.0 to 2.5 µm are obtained. Note that after the acid etching and cleaning processes, an electronegative MXene surface can be easily achieved due to the existence of abundant terminal groups (i.e., —OH, —O, and —F), which facilitates the electrostatic absorption of zinc cations (Zn^2+^) for in situ growth of ZIF‐L. The absorbed metallic ions acting as nucleation sites can quickly coordinate with 2‐MeIM and crystallize to generate flower‐like ZIF‐L particles with perpendicularly‐aligned petals. As shown in the SEM and TEM images of the bio‐inspired hierarchical ZIF‐L@Ti_3_CNT*_x_* composites (Figure 2g,h), the ZIF‐L particles with a structure and size similar to those of pristine ones are firmly and randomly in situ assembled on the MXene nanosheets. In addition, the spatial distribution of the Ti, C, N, O, F, and Zn elements in the ZIF‐L@Ti_3_CNT*_x_* composites is confirmed in the corresponding elemental mapping (Figure 2i). The above results well prove the successful fabrication of ZIF‐L@Ti_3_CNT*_x_* composites. It is worth mentioning that the introduction of flower‐like ZIF‐L particles can forcefully suppress the electrostatic repulsion between the Ti_3_CNT*_x_* nanosheets; thus, effectively alleviating the self‐restacking of MXene nanosheets.^[^ ^10a^ ^]^ Moreover, the integration of ZIF‐L particles and Ti_3_CNT*_x_* nanosheets promotes the formation of ionic or electronic transportation pathways and improves accessibility of active sites.^[^ ^14^ ^]^

**Figure 3:**
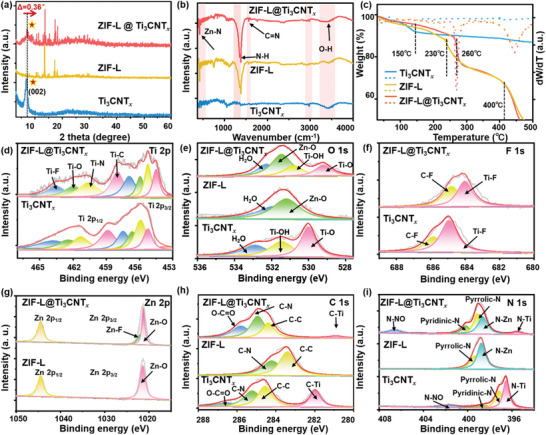
a) XRD patterns, b) FTIR, and c) TGA profiles of Ti_3_CNT*_x_* nanosheets, ZIF‐L particles, and ZIF‐L@Ti_3_CNT*_x_* composites. The XPS profile of the d) Ti 2p, e) O 1s, f) F 1s, g) Zn 2p, h) C 1s, and i) N 1s orbits in different Ti_3_CNT*_x_* nanosheets, ZIF‐L particles, and ZIF‐L@Ti_3_CNT*_x_* composites.

To investigate the structure of the as‐synthesized composites, X‐ray diffraction (XRD) patterns of the ZIF‐L@Ti_3_CNT*_x_* composites, the pristine Ti_3_CNT*_x_* nanosheets, and ZIF‐L particles are conducted (**Figure** **3**a; Figure S3, Supporting Information). Consistent with the obtained HRTEM and SAED results, the Ti_3_CNT*_x_* nanosheets exhibit single phase of characteristic (002) diffraction peaks (2*θ* = 6.78°), confirming the successful exfoliation of delamination.^[^ ^15^ ^]^ The diffraction peaks of ZIF‐L particles are in good agreement with the theoretically simulated result of the ZIF‐L.^[^ ^16^ ^]^ In the ZIF‐L@Ti_3_CNT*_x_* composite, the typical diffraction peaks of both Ti_3_CNT*_x_* and ZIF‐L particles are observed, suggesting that the in situ growth of MOF particles did not disrupt the structural integrity of the 2D Ti_3_CNT*_x_* nanosheets. The weak (002) diffraction peak of the ZIF‐L@Ti_3_CNT*_x_* composite is caused by some small splintered Ti_3_CNT*_x_* nanosheets generated from in situ growth of ZIF‐L via sonication. Similar attenuation phenomenon of (002) diffraction peak is often observed in the MXene based composites.^[^ ^10^, ^17^ ^]^ Notably, the characteristic (002) peak of MXene in the ZIF‐L@Ti_3_CNT*_x_* composites is found to slightly upshift to a higher angle (2*θ* = 7.14°), which is related to the contraction of *d*–spacing in MXene after perpendicular assembly with MOF particles.^[^ ^11^ ^]^ The chemical structure transformation is further verified using Fourier transform infrared spectroscopy (FTIR, Figure 3b). For the Ti_3_CNT*_x_* nanosheets, the stretching peaks at 3000 and 3443 cm^−1^ mainly attribute to the N—H and O—H stretching modes of the surface terminal functional groups. Additional vibration peaks at 420 cm^−1^ (Zn—N) and 1581 cm^−1^ (C=N) appear in the ZIF‐L@Ti_3_CNT*_x_* composites in comparison with the pristine Ti_3_CNT*_x_* nanosheets, indicating the successful composition of the Ti_3_CNT*_x_* nanosheets and ZIF‐L particles.^[^ ^10a^ ^]^

Thermogravimetric analysis (TGA) and differential thermogravimetric analysis (DTG) curves are displayed in Figure 3c to investigate the thermal stability of as‐synthesized samples. In the Ti_3_CNT*_x_* nanosheets, the slight weight loss between 100 °C and 150 °C is ascribed to the removal of physically adsorbed water and gases, that is, O_2_. The gradual weight loss between 150 °C to 500 °C is related to the wiping of the chemically adsorbed —OH groups on the Ti_3_CNT*_x_* surface.^[^ ^18^ ^]^ The 4.84 wt% weight loss in this temperature range proves —OH groups enriched surface, which contributes to the mutual grafting and recombination of heterogeneous functional materials. Both the ZIF‐L particles and ZIF‐L@Ti_3_CNT*_x_* composites exhibit similar attenuation trends in their TGA curves. The first descending step below 230 °C is related to the removal of physically adsorbed water and O_2_. A slight curve attenuation exists in the temperature ranging of ≈230–400 °C for the ZIF‐L particles and 260–400 °C for the ZIF‐L@Ti_3_CNT*_x_* composites, ascribed to the removal of chemically adsorbed water. The last stage of weight loss in the range of 400–500 °C is attributed to the decomposition of the organic linker (2‐MeIM) in the ZIF‐L framework, which is consistent with a previous study.^[^ ^19^ ^]^ TGA analysis confirmed that the ZIF‐L@Ti_3_CNT*_x_* composites structure is stable before 260 °C, which provides guidance for the subsequent gas‐sensing test at room temperature (RT).

X‐ray photoelectron spectroscopy (XPS) was utilized to characterize the chemical composition of the Ti_3_CNT*_x_* nanosheets, ZIF‐L particles, and ZIF‐L@Ti_3_CNT*_x_* composites. As illustrated in Figure 3d, the existence of Ti—C (455.2 /458.8 eV) and Ti—N (455.9 /461.3 eV) in the Ti_3_CNT*_x_* nanosheets indicates that a few layers Ti_3_CNT*_x_* is successfully fabricated. In addition, the as‐prepared Ti_3_CNT*_x_* nanosheets carry many surface functional groups after selective‐etching and delamination of the parent Ti_3_AlCN phase, which can be verified by the existence of the Ti—O (456.6/462.4 eV) and Ti—F (457.5/463.8 eV).^[^ ^15^ ^]^ These abundant terminal organic functional groups are further observed in the O 1s and F 1s XPS peaks (Figure 3e,f). Owing to the formation of Ti—O—Zn and Ti—F—Zn after in situ growth of ZIF‐L particles, the peak area proportions of Ti—O and Ti—F peaks in ZIF‐L@Ti_3_CNT*_x_* composites decrease, as exhibited in Table S1, Supporting Information. This can be further confirmed by the appearance of Zn—O in the O 1s (531.4 eV in Figure 3e) and the Zn—F bond in the Zn 2p XPS peaks (1021.9 eV in Figure 3g) in the ZIF‐L@Ti_3_CNT*_x_* composites, illustrating that ZIF‐L particles and Ti_3_CNT*_x_* nanosheets are connected via Ti—O—Zn and Ti—F—Zn chemical bridge. Furthermore, the difference in the C 1s, N 1s, and O 1s XPS peaks of the ZIF‐L particles and ZIF‐L@Ti_3_CNT*_x_* composites provide further detailed evidence for their electrostatic assembly. For the ZIF‐L particles, there are only two C 1s peaks at 284.6 and 285.5 eV, corresponding to the C—C and C—N bonds, respectively (Figure 3h). Two additional C 1s peaks ascribed to the C—Ti (280.9 eV) and O—C=O bonds (285.8 eV) of the Ti_3_CNT*_x_* nanosheets appear in the ZIF‐L@Ti_3_CNT*_x_* composites. This indicates the successful combination of the Ti_3_CNT*_x_* nanosheets and the ZIF‐L particles. The similar XPS peaks coupling phenomenon can also be further discovered in the O 1s and N 1s spectra in Figure 3e,i. In addition, as compared to the Ti_3_CNT*_x_* nanosheets, the binding energies of Ti 2p, C 1s, O 1s, and F 1s peaks in ZIF‐L@Ti_3_CNT*_x_* composites shift to the lower binding energy side, which result from additional electron density as integrated with the ZIF‐L particles with the Ti_3_CNT*_x_* nanosheets surface. Combining the above results, it can be confirmed that the ZIF‐L particles were firmly grafted to the MXene surface via the Ti—O—Zn and Ti—F—Zn bonds, resulting in good interfacial adhesion for superior electron/ion conduction.

### 2.2 DMA Gas Sensing Properties of ZIF‐L@Ti_3_CNT*_x_* Composites Based Flexible Sensors

**Figure 4:**
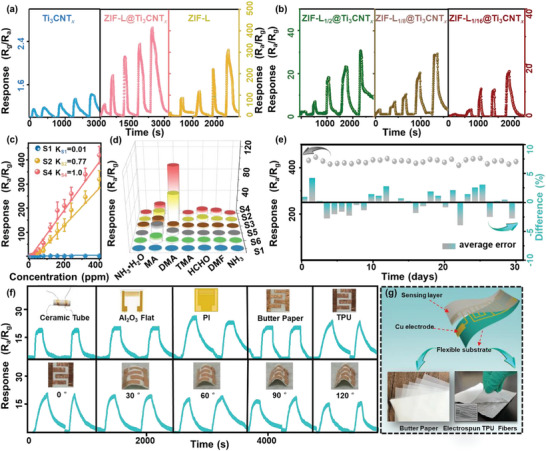
The dynamic response of various sensors to the 80–400 ppm of dimethylamine (DMA) gas at RT, including the a) Ti_3_CNT*_x_*, ZIF‐L, and ZIF‐L@Ti_3_CNT*_x_* (ZIF‐L_1/4_@Ti_3_CNT*_x_*) sensors, and b) ZIF‐L_1/2_@Ti_3_CNT*_x_*, ZIF‐L_1/8_@Ti_3_CNT*_x_*, and ZIF‐L_1/16_@Ti_3_CNT*_x_* sensors, respectively. c) The linear relationship of the Ti_3_CNT*_x_*, ZIF‐L@Ti_3_CNT*_x_*, and ZIF‐L sensors to 1–400 ppm of the DMA gas. d) The selectivity to 100 ppm of various VOCs and e) long‐term stability tests of the ZIF‐L@Ti_3_CNT*_x_* sensors to 400 ppm DMA gas. f) Various responses of the ZIF‐L@Ti_3_CNT*_x_* sensors based on different electrodes (i.e., ceramic tube electrodes, ceramic planer interdigital electrodes, PI substrate with Au electrodes, butter paper, and TPU substrates with Cu electrodes as shown in the insets) to 55 ppm DMA gas, and the response of the ZIF‐L@Ti_3_CNT*_x_* sensors based on TPU electrode which bent at different angles (from 0–120°; bottom of the picture). g) The schematic diagram of the flexible ZIF‐L@Ti_3_CNT*_x_* sensors structure.

Ingenious biomimetic structures provide ideal templates for building high‐performance sensing materials, endowing them with flexible architectures and advanced functions.^[^ ^20^ ^]^ Herein, a flexible gas sensor is fabricated using biomimetic ZIF‐L@Ti_3_CNT*_x_* composites which is inspired by the biological synapse structure and anticipated to obtain enhanced sensitivity to trace amounts of the DMA exhaled biomarker of Parkinson's disease. **Figure** **4**a displays the dynamic response signals of the Ti_3_CNT*_x_*, ZIF‐L, and the ZIF‐L@Ti_3_CNT*_x_* sensors to 80–400 ppm DMA gas at RT. The dynamic curves of the ZIF‐based sensors show a reduction in resistance upon injection of DMA gas (Figure S4, Supporting Information) with response value defined as *R*_a_ */R*_g_. In comparison, the Ti_3_CNT*_x_* sensor only shows an increase in resistance with response value defined as *R*_g_ */R*_a_ and a very low response signal to DMA gas (*R*_g_ */R*_a_ = 1.2 ± 0.02 to 80 ppm), indicating the ZIF‐L particles play a dominant role in detection of DMA gases in the ZIF‐L@Ti_3_CNT*_x_* sensors. However, after combining with the ZIF‐L particles, the response of the ZIF‐L@Ti_3_CNT*_x_* sensors to DMA gas is significantly improved (*R*_a_ */R*_g_ = 55±8), which is also higher than that of the ZIF‐L sensors (*R*_a_ */R*_g_ = 20±6). Accordingly, the sensitivity of the ZIF‐L@Ti_3_CNT*_x_* sensors is further optimized by modulating the molar ratio of Zn^2+^ metallic precursor and 2‐MeIM ligands to obtain different structure of ZIF particles. As shown in Figure S5, Supporting Information, as the molar ratio of Zn^2+^ metallic precursor and 2‐MeIM ligands increase from 1:2 to 1:16, the number of petals of ZIF‐L particles gradually increase. The significant difference in the morphology of the ZIF particles is ascribed to the secondary nucleation and growth of the initial ZIF‐L petals.^[^ ^21^ ^]^ The dynamic sensing tests of the ZIF‐L@Ti_3_CNT*_x_* sensors with four different molar ratios of the Zn^2+^ metallic precursor and 2‐MeIM ligands are shown in Figure 4b; Figure S6, Supporting Information. The ZIF‐L_1/4_@Ti_3_CNT*_x_* sensors exhibit the highest response to the DMA gas. Thus, the subsequent sensing performance study of the composites mainly focuses on the ZIF‐L_1/4_@Ti_3_CNT*_x_* sensors, which are marked as the ZIF‐L@Ti_3_CNT*_x_* sensors for convenience.

The linear correlations between the response and DMA concentration (1–400 ppm) of the Ti_3_CNT*_x_*, ZIF‐L, and the ZIF‐L@Ti_3_CNT*_x_* sensors are further shown in Figure 4c. The slope of the calibration curve of the ZIF‐L@Ti_3_CNT*_x_* sensors is the highest compared to those of the other two sensors. According to the dynamic response curves (Figure 4a), the response/recovery times of the ZIF‐L@Ti_3_CNT*_x_* sensors to 240 ppm DMA gas are calculated. The response and recovery times of the ZIF‐L@Ti_3_CNT*_x_* sensors (36 s / 170 s) are much lower than those of the Ti_3_CNT*_x_* (68 s / 285 s) and ZIF‐L sensors (120 s / 227 s) (Figure S7, Supporting Information). This rapid dynamic response process is ascribed to the incorporation of conductive Ti_3_CNT*_x_*, which significantly shortens the ion migration distance by generating extensive inter‐connected hydrogen networks.^[^ ^12a^ ^]^ In particular, the hierarchical assembly of ZIF‐L particles and Ti_3_CNT*_x_* nanosheets favors the accessibility of DMA gas, which greatly shortens the transport and diffusion distances of protons, thereby achieving the fastest response. Table S2, Supporting Information, compares the sensing parameters of recently reported MXene and ZIF‐based gas sensors with those of the ZIF‐L@Ti_3_CNT*_x_* sensor. Benefiting from the reasonable bionic strategy, the response of the ZIF‐L@Ti_3_CNT*_x_* sensor is noticeably higher than that of most of the surveyed sensors with satisfied response/recovery times.

Good selectivity is an important sensing indicator for practical detection. Figure 4d shows that the chemiresistive response of the ZIF‐L@Ti_3_CNT*_x_* sensors to a range of alkalines with similar molecular structure is comparatively shown in, including 100 ppm of HCHO, DMF, MA (CH_3_NH_2_), DMA (C_2_H_7_N), TMA (C_3_H_9_N), NH_3_·H_2_O, and dry NH_3_ gases. Notably, a significantly enhanced selectivity compared to interfering gases (a 2‐fold improvement) and the other kind of gas sensors (a 4.6‐fold improvement) is successfully achieved in the optimal ZIF‐L@Ti_3_CNT*_x_* sensors to DMA gas. The amino groups in DMA facilitate the formation of hydrogen‐bond networks with the imidazole molecules of ZIF‐L particles; thus, improving the DMA sensing properties.^[^ ^22^ ^]^ In addition, the long‐term stability of the ZIF‐L@Ti_3_CNT*_x_* sensor is also investigated. As shown in Figure 4e, the ZIF‐L@Ti_3_CNT*_x_* sensors can maintain 93.4% of the original response after storage in the ambient environment for 30 days, which exhibits only a slight fluctuation (<5%) from the average response. The excellent long‐term stability is related to the good structural stability, as proven in previous TGA analysis, as well as the water stability of ZIF‐L sensing materials because they are fabricated in aqueous solution. Besides, the assembly of ZIF‐L particles can also effectually avoid the self‐stacking and oxidation occurring in the Ti_3_CNT*_x_* nanosheets layers. The above properties endow the optimal ZIF‐L@Ti_3_CNT*_x_* sensors based on biomimetic synapse‐like structure with robust long‐term durability in complicated practical applications.

To realize the versatile applications in various scenarios, it is imperative to develop the universality and compatibility of sensing materials on different sensing substrates. Specifically, the compatibility of the bio‐inspired ZIF‐L@Ti_3_CNT*_x_* composites is investigated by modifying them on various electrodes, from rigid to flexible substrates. Figure 4f exhibits the dynamic sensing curves to 55 ppm DMA gas of ZIF‐L@Ti_3_CNT*_x_* sensors on the ceramic tube, ceramic planer interdigital electrode, flexible PI substrates with Au electrodes, self‐made butter paper, and TPU substrates with Cu electrodes. The preparation process of the self‐made butter paper and electro‐spun TPU substrates is illustrated in Figure 4g. The response values of various sensors, shown in Figure 4f, are summarized in Figure S8, Supporting Information, and their relative standard deviation (RSD) of the response is calculated to be 12.8%. Specifically, the response signals of flexible electrodes are slightly higher than those on the rigid substrates, ranging from 18.2 ± 0.02 of the ceramic tube electrode to 24.8 ± 1.5 of the PI electrode. Especially, the response of the sensor on home‐made TPU flexible electrode to 55 ppm DMA gas (*R*_a_ */R*_g_ = 23.1±0.2) is almost identical to that of sensors on the commercial PI electrodes, suggesting a superior applicability of TPU substrate. These results demonstrate the universality of the ZIF‐L@Ti_3_CNT*_x_* composites on various substrates. To further investigate the flexibility and feasibility, the binding test of ZIF‐L@Ti_3_CNT*_x_* sensors based on the TPU electrode (0–120°) is performed by recording the responses to 55 ppm DMA gas at different angles. The response of the sensors under diverse bending angles shows only a slight change, with an RSD of 9.6%. The response value bent at 120° (*R*_a_ */R*_g_ = 17.9±0.5) can maintain 77.5% of the original response value (*R*_a_ */R*_g_ = 23.1±0.3; Figure S9, Supporting Information). These advances make the flexible ZIF‐L@Ti_3_CNT*_x_* sensor very promising for applications in wearable electronics for the detection of DMA gas in real application scenarios.

### 2.3 DMA Sensing Mechanism of the Bio‐Inspired ZIF‐L@Ti_3_CNT*_x_* Sensors

**Figure 5:**
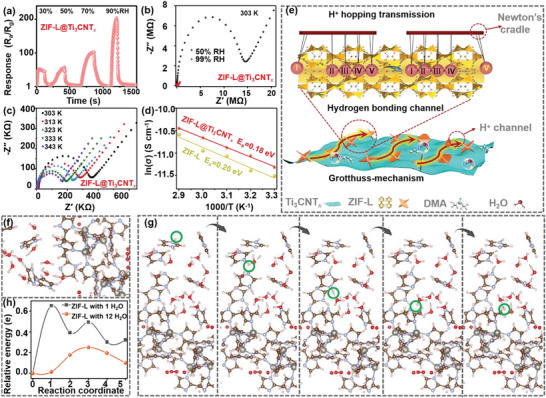
a) Various responses of the ZIF‐L@Ti_3_CNT*_x_* sensors to 80 ppm DMA under various RH (30–90%) at 303 K. Nyquist plots of the ZIF‐L@Ti_3_CNT*_x_* sensors to 100 ppm DMA at 303 K under b) 50% and 99% RH and c) at 303–343 K under 99% RH. d) Arrhenius plots of the ZIF‐L@Ti_3_CNT*_x_* and ZIF‐L sensors at 303–343 K under 99% RH. e) The schematic illustration of the proton transportation mechanism of the ZIF‐L@Ti_3_CNT*_x_* sensors. f) The structural configurations and g) DFT calculations of the proton‐binding sites during relaxation of ZIF‐L placed with 12 water molecules, where the proton is highlighted by the green circle. h) The activation energy barrier for the proton transfer in ZIF‐L structure with 12 and 1 water molecules; the reaction path is obtained by climbing image NEB (using five images); the abscissa and ordinate represent the path of proton and the energy barrier in the transport process, respectively.

Constructing the bionic structure is not only aimed to the similarity in shape but also to the working mechanism, which is key for the improving device performance. The unique ions transferring mechanism of biological synapses endow the biomimetic electronic devices with merits of high‐sensitivity, high‐speed, and strong specificity, which offer inspirations for achieving more realistically bionic function.^[^ ^6a^ ^]^ This is another reason for choosing ZIF‐L particles in bio‐inspired ZIF‐L@Ti_3_CNT*_x_* composites because they can exhibit a proton conduction mechanism similar to that in the synapse.^[^ ^23^ ^]^ To confirm the proton conduction mechanism, the response of the Ti_3_CNT*_x_*, ZIF‐L, and ZIF‐L@Ti_3_CNT*_x_* sensors to 80 ppm of dry DMA gas (without H_2_O molecules) is measured. As shown in Figure S10, Supporting Information, almost no response signals are observed. However, the responses of the three sensors to 80 ppm DMA gas gradually rise with increasing the humidity (**Figure** **5**a; Figure S11, Supporting Information). Last, a 3.6‐fold enhanced response of the ZIF‐L@Ti_3_CNT*_x_* sensors is obtained as the humidity is increased from 35% to 90%, confirming the essential role of water molecules in the DMA gas sensing process. Therefore, DMA gas is prepared by the evaporation of the DMA solution (40 wt%, AR) via the static volumetric method in all sensing tests, unless otherwise stated. These properties make ZIF‐L@Ti_3_CNT*_x_* sensor highly suitable for the detection of breath markers in high‐humidity environments. The humidity calibration curves of the Ti_3_CNT*_x_*, ZIF‐L, and ZIF‐L@Ti_3_CNT*_x_* sensors are established under various RH (30–90%) by executing varying humidity response tests, respectively. As presented in Figure S12, Supporting Information, when exposed to the same 80 ppm of DMA gas under different RH ambient, the differences between the total response and humidity response almost keep consistent in both of ZIF‐L and ZIF‐L@Ti_3_CNT*_x_* sensors. Therefore, the detection deviation introduced by various humidity levels can be obviated by calculating the difference between these two values when necessary.

In addition, alternating‐current impedance measurements are conducted to monitor the increases in conductivity with humidity to verify the proton conduction behavior. The proton conductivity values (*σ*) are calculated using Equation (S2), Supporting Information, according to Figure 5b; Figure S13 and Table S3, Supporting Information. The *σ* value of ZIF‐L@Ti_3_CNT*_x_* composites at 99% RH increases 38.7 times than the value at 50% RH, which pertains to typical proton conduction behavior.^[^ ^10b^ ^]^ It is higher than the conductivity variation of the ZIF‐L particles (34.5 times). In contrast, the change of the *σ* value of Ti_3_CNT*_x_* nanosheets obeys an opposite trend, indicating a different sensing mechanism, which is consistent with previous studies.^[^ ^11^ ^]^ To further confirm the exact type of the proton conduction mechanism, the activation energy (*E*_a_) values of the ZIF‐L@Ti_3_CNT*_x_* composites and ZIF‐L particles are calculated via the Arrhenius equation: Equation (S3), Supporting Information, according to the temperature‐dependent proton conducting behaviors (Figure 5c; Figure S14 and Table S4, Supporting Information). As calculated, *E_a_* values of the ZIF‐L@Ti_3_CNT*_x_* composites and ZIF‐L particles are determined to be 0.18 and 0.20 eV, respectively (Figure 5d). Generally, if the *E*_a_ value ranges in 0.1–0.4 eV, it indicates that the the proton transfer process is controlled by the Grotthuss mechanism.^[^ ^10b^ ^]^ The lower *E*_a_ value of the ZIF‐L@Ti_3_CNT*_x_* composites facilitates the proton hopping, which can reasonably explain why the ZIF‐L@Ti_3_CNT*_x_* composites sensors exhibited the highest response to DMA gas.

To further elucidate the proton migration pathway in the hydrogen‐bonding network, density functional theory (DFT) simulation is performed. As the sensing response is dominated by the ZIF‐L particles in the bio‐inspired ZIF‐L@Ti_3_CNT*_x_* composites, it is considered as the object for in‐depth theoretical calculation. Two types of stable structural configurations of ZIF‐L particles exposed to the environment with and without water molecules are displayed in Figure 5f; Figure S15, Supporting Information, respectively. The proton migration pathway of DMA/ZIF‐L system is built by randomly placing 12 water molecules to simulate the wet DMA gas atmosphere. As shown in Figure 5g, a foreign proton bonded to the N site without H in the imidazole molecule can cause the H atom on the adjacent N site to dissociate. The as‐dissociated H^+^ then interacts with the neighboring water molecule, further causing the water molecule to remove one H^+^. Then, the detached H^+^ as a trigger agent reacts again with the N sites in the imidazole molecule nearby, which leads to the next round of the proton insertion and de‐insertion process via the breakage and reconnection of hydrogen bonds. As compared, the proton hopping migration in DMA/ZIF‐L system with few water molecules (one water) occurs between the imidazole molecules. As shown in Figure S16, Supporting Information, owing to the absence of a sufficient quantity of water molecules in this system, only the initial proton and subsequent imidazole binding sites participate in constituting the H transferring paths. This process restricts the transfer and hopping of proton and results in a lower proton conductivity, as confirmed in Figure 5b. To confirm the proton transfer capacity in these two different pathways, the climbing image‐nudged elastic Band (Cl‐NEB) method is further employed to compute the activation energy of the proton hopping. The activation energy barrier of DMA/ZIF‐L system with 12 water molecules is estimated as 0.25 eV which is close to the experiment value (0.16 eV, Figure 5h), unambiguously illuminating a Grotthuss proton conduction mechanism. The discrepancy in *E*_a_ values is related to the difference in the concentrations and mobility of the proton carriers between the experiments and computational simulation.^[^ ^22^ ^]^ In addition, the DMA/ZIF‐L system with a few water molecules exhibits a much higher *E*_a_ value (0.65 eV), indicating a harder pathway for protons transferring. The above results further identify the crucial role of the water molecules during protons hopping in the ZIF‐L sensing system. As schematically described in Figure 5e, when incorporated with Ti_3_CNT*_x_* nanosheets, the abundant absorbed water molecules in the hydrophilic channels and terminal functional groups (—F, —OH, and —O) of the Ti_3_CNT*_x_* nanosheets surface can strongly assist to form hydrogen‐bond networks to promote proton transport.^[^ ^12a^ ^]^ Combining the excellent proton conductivity capacity of ZIF‐L particles and long‐range continuous proton transfer pathways on the surface of the Ti_3_CNT*_x_* nanosheets, the ZIF‐L@Ti_3_CNT*_x_* composites obtain accelerated proton mobility and enhanced DMA gas sensing properties. The sensing performance of the sensors is closely related to the types and amounts of terminal groups of the MXene surface. To further evaluate the effect of terminal groups of the Ti_3_CNT*_x_* surface on the dual‐mode DMA gas and strain sensor, the amount of −OH of the Ti_3_CNT*_x_* was increased by LiOH alkalization treatment.^[^ ^24^ ^]^ As shown in Figure S17, Supporting Information, compared to the pristine Ti_3_CNT*_x_* nanosheets, the peak area proportions of −OH in O 1s XPS spectra significantly increase after LiOH treatment (labeled as Ti_3_CNT_OH_). This demonstrates that the alkalization treatment endows the Ti_3_CNT*_x_* surface rich in −OH terminals. In addition, the DMA gas sensing properties of both pristine ZIF‐L@Ti_3_CNT*_x_* and ZIF‐L@Ti_3_CNT_OH_ sensors are explored, respectively. The response of ZIF‐L@Ti_3_CNT_OH_ sensors to 100 ppm DMA is higher than that of ZIF‐L@Ti_3_CNT*_x_* sensors, related to increased —OH being able to form more hydrogen bond networks in MXene surface to facilitate the proton transport.

### 2.4 Strain‐Sensing Performance of Flexible ZIF‐L@Ti_3_CNT*_x_* Sensors

**Figure 6:**
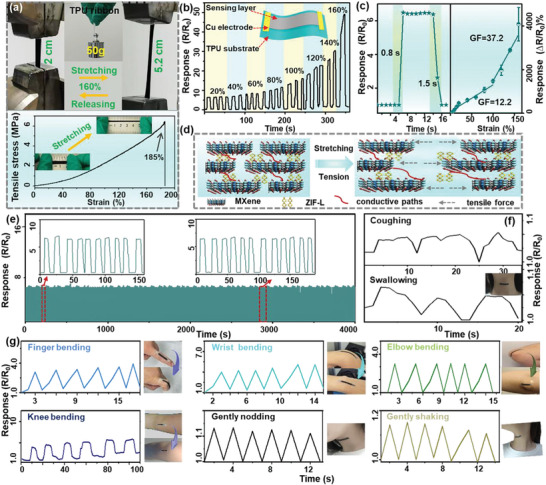
Stretching performance of the flexible ZIF‐L@Ti_3_CNT*_x_* strain sensors. a) The optical images of the strain sensor before (left) and after (right) stretching, and the tension changes of the sensor under different stretching strains. b) Dynamic response curves of the strain sensor with various stretching variables (from 20% to 160%; inset shows the photo of the strain sensor). c) The calculated response/recovery times of the ZIF‐L@Ti_3_CNT*_x_* sensors to 20% stretching deformation (left) and the corresponding GF (right). d) Schematic sensing mechanism of the electromechanical sensor. e) Repeatable response curves for 400 cycles of stretching/relaxing. f) Resistance‐signal response of throat muscle movement such as coughing and swallowing. g) The resistance‐signal response of finger, wrist, elbow, knee bending, and head movement patterns including gently nodding and shaking.

Benefiting from the well‐designed bionic architectures, excellent humidity assisted ionic conductivity, as well as the excellent universality on multiple types of electrodes, the ZIF‐L@Ti_3_CNT*_x_* composites provide an excellent choice for designing a new physiological movement monitoring platform, especially on the sweaty skin. Herein, the ZIF‐L@Ti_3_CNT*_x_* composites are fabricated as a flexible strain sensor via coating on the flexible and permeable TPU membrane attached with a pair of copper electrodes, and the size of strain sensor is 20 × 5 × 0.13 mm ^3^. A large tensile range is one of the most vital indicators for the strain sensing property in practical use.^[^ ^1a^ ^]^ The chosen electrospun TPU nanofiber substrate is lightweight and highly stretchable, which can lift 50 g weight without any deformation (inset of **Figure** **6**a); thus, guaranteeing the remarkable durability of the fabricated strain sensor. The left and right pictures in Figure 6a show that the strain sensor can sustain a wide range of 0–160% of tension with a reliably stretchable strength of 6.5 MPa, which facilitates to monitor deformation signals generated by body movements.

The resistance variation of the ZIF‐L@Ti_3_CNT*_x_* strain sensors in wetting conditions under various cyclic stretching/relaxing is measured to investigate its electromechanical properties. Dramatically different from the decreasing trend of the sensor resistance in response to DMA gas, the strain sensor with the same ZIF‐L@Ti_3_CNT*_x_* composites exhibits an increasing tendency of the resistance as triggered by tension (Figure S18, Supporting Information). This nonoverlapping strain and gas response behavior are beneficial for building the dual‐mode sensing platform based on ZIF‐L@Ti_3_CNT*_x_* composites.^[^ ^25^ ^]^ In addition, the response/recovery times of the sensor are determined to be 0.8/1.5 s under 20% deformation (Figure 6c). As shown in Figure S19, Supporting Information, when the ZIF‐L@Ti_3_CNT*_x_* sensor was exposed to 1 ppm DMA gas, a relatively slow response process could be detected. However, as further applied 40% strain deformation with the existence of DMA gas, the resistance of sensor was immediately increased. This is because increased distance of conductive pathways by stretching can cause a sudden contact separation of conductive materials. Gauge factor (GF) defined as |*∆R/R*_0_|/∆*ε* is employed to evaluate the sensitive performance of the tensile sensor, where ∆*R* represents the resistance variation before and after applying tension, *R*_0_ is the initial resistance without tension, and ∆*ε* is the applied strain variation.^[^ ^1a^ ^]^The GF of strain sensor is closely dependent on the diverse tension. As shown in Figure 6c, it exhibits a slow increase in a relatively small strain below 100% with sensitivity of 12.2; then, follows by a sharp increase (GF of 37.2) under strain changing from 100–160%. As compared with previous works (Table S5, Supporting Information), the GF of the ZIF‐L@Ti_3_CNT*_x_* sensor in this work is higher than those of most strain sensors, indicating an excellent sensitivity to diverse intensity of stretching deformation. In the low‐strain range, the distance and space of proton conducting pathways increased under stretching deformation; thus, causing a small resistance transition and low sensitivity. However, the contact space and interconnecting points of conducting pathways significantly decrease as a larger tensile is loaded, resulting in a sharply increased resistance (Figure 6d). Importantly, the hydrogen bonds networks in the ZIF‐L@Ti_3_CNT*_x_* strain sensors can effectively prevent the fracture of conductive paths when a larger external force is introduced. Therefore, the response remains rapid and stable even during 400‐times cyclic tests of stretching/relaxation (Figure 6e). In addition, the actual detection limit of the stretching strain sensor is determined to as low as 5% (Figure S20, Supporting Information), illustrating the potential to perceive micro tremor of the human skin. The strain sensor based on pristine Ti_3_CNT*_x_* nanosheets exhibits a lower response to 40% stretching deformation as compared to that of ZIF‐L@Ti_3_CNT*_x_* strain sensor (Figure S21, Supporting Information). Similarly, the stretching sensing performance of ZIF‐L@Ti_3_CNT_OH_ sensor is also improved after LiOH treatments (Figure S22, Supporting Information). Further studies can be carried out to investigate the detailed influence of surface groups of MXene on the sensing performance based on the proton conduction device.

To evaluate the capability to monitor the physiological motion, the ZIF‐L@Ti_3_CNT*_x_* strain sensors are adhered to various joint parts of the body. As exhibited in Figure 6f, the as‐prepared strain sensor is first attached to the throat of a volunteer. A clear and reproducible response of the strain sensor to the subtle deformation signals of the epidermis and muscle is observed, such as coughing and swallowing. In addition, the permeable electro‐spun TPU flexible substrate is easier to absorb sweat secreted in the skin. High humidity substrate interface will further enhance ionic conductivity of the ZIF‐L@Ti_3_CNT*_x_* strain sensors and promote the perception of human motion signals. When the strain sensor is adhered onto finger, wrist, elbow, and knee joints, respectively (Figure 6g), it can quickly exhibit characteristic stretching and relaxing peaks in response to larger tensile deformations. Moreover, the exceptional sensitivity, stretchability, and stability enables the strain sensor to accurately recognize the manner of the head motion such as gently shaking and nodding one's head. These results demonstrate the excellent ion conducting and mechanical performances of ZIF‐L@Ti_3_CNT*_x_* strain sensors, making them promising for real‐time, wearable health‐assessment, and remote medicine diagnosis. Furthermore, excellent compatibility with a thinner flexible TPU substrate and robust stretchability render the strain sensor much easier to attach to human skin; thus, significantly reducing signal scatter.

### 2.5 Wearable and Smart Health‐Monitoring Application Using Flexible Gas and Strain Sensors

**Figure 7:**
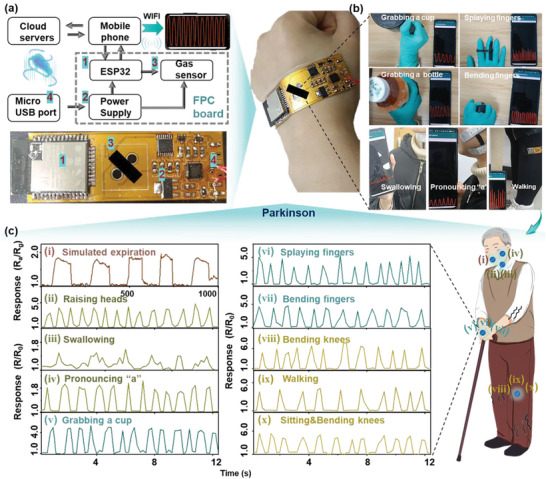
Flexible and smart wearable healthcare system for the detection of physiological signals of Parkinson. a) Schematic illustrations of the dual‐mode signal acquisition and display system including signal acquisition, processing, wireless data transmission by WIFI, Alibaba cloud storage, and mobile display (functional unit components are labeled in the circuit diagram). b) Photograph of a flexible circuit that is attached on various parts of the body to track human motions. c) The real‐time response of the flexible sensors to c‐i) the simulated exhalation and c‐ii–iv) head, c‐v–vii) hands, and c‐viii–x) legs movements.

Benefiting from the bio‐inspired structure, the ZIF‐L@Ti_3_CNT*_x_* sensors can accurately detect DMA gas with the assistance of humidity in the form of resistance decrease, while also perceiving variation of stresses in the form of resistance increase. Note that specific markers of DMA in exhaled breath and abnormal body tremors, such as stilly shacking, bradykinesia, rigidity muscles, and abnormal posture and pace, are considered early warning signs of Parkinson's disease.^[^ ^2a^ ^]^ These properties make it exhibit great potential in reliable medical monitor for both exhalation detection and body motion healthcare management. Accordingly, an intelligent and flexible circuit is designed by integrating the as‐fabricated flexible ZIF‐L@Ti_3_CNT*_x_* gas and strain sensors on a removable terminal. The module components and circuit diagrams of the smart device are shown in **Figure** **7**a, respectively, including the component units of signal acquisition, processing, WIFI wireless data transmission, Alibaba cloud storage, and mobile display. The collected physiological signals are uploaded in real‐time to the cloud to establish electronic health (e‐health) archives, which contribute to improving the telemedicine monitoring system, especially for patients with Parkinson's disease. The boundary size of the flexible device is only 5 × 2 cm^2^. Detailed information on the flexible circuit is provided in Supporting Information. Figure 7b shows photographs of representative sensing scenes, in which the flexible circuit is attached to various parts of the body to track human motions. As expected, the ZIF‐L@Ti_3_CNT*_x_* sensor could respond to 1 ppm DMA gas in the exhalation background with rapid response and recovery dynamics (panel (i) in Figure 7c; Figure S23 and Movie S1, Supporting Information). In addition, the sensor can also be fixed onto the skin surface to discriminate the physical motion postures. The panels (ii–iv) in Figure 7c exhibit a very stable resistance transition when the sensor is mounted onto the throat to recognize a weak strain deformation, such as raising one's head, swallowing, and pronouncing, respectively (Figure S24 and Movie S2, Supporting Information). The panels (v–vii) show the resistance variation of the flexible sensor when settled onto the fingers (Figure S25 and Movie S3, Supporting Information). It is clearly observed that the resistance varies closely depending on the degree of exertion when grabbing the cup, splaying, and bending the fingers in varying amplitudes. A significant resistance change happens when the sensor is fixed on the knee joints to identify the body sporting posture, such as knee bending, walking and sitting, and bending one's knees ((viii–x) in Figure 7c; Figure S26 and Movie S4, Supporting Information). In short, the flexible sensor can always immediately respond to a variety of stimuli strain signals. Moreover, the flexible sensor shows a high sensitivity, reproducible stability, and durability to both exhaled biomarker and body kinematics signals. Specially, it displays totally different variation direction of resistance upon applying DMA gas or tension, guaranteeing an accurate differentiation to varieties of physical stimulations. To further evaluate the detection accuracies of the proposed sensors, 20 cycling tests of the simulated exhalation and bending movements of knee were performed, respectively. In the data group of exhaled breath, 16 of all the 20 tested results showed similarity with other four data lower than the normal response values (Figure S27, Supporting Information). The accuracy of the ZIF‐L@Ti_3_CNT*_x_* sensor in DMA gas sensing mode is therefore estimated to be 80%. Similarly, the accuracy in strain‐sensing mode is estimated to 90%. In addition, the intelligent wearable system can still output stable DMA gas and strain detection signals after bending the flexible circuit board for 50 times, further indicating a good effectiveness and working life (Figure S28, Supporting Information). These results indicate that the proposed dual–mode ZIF‐L@Ti_3_CNT*_x_* sensor demonstrates a feasible and potential application in recording body coordination movement for the Parkinson patients. Although the as‐fabricated bionic sensors can sensitively recognize human motion information, other more multi‐complex motion features, such as attitude angle matching recognition, multiple simultaneous monitoring of temperature, humidity, gas, and motion signals, are still facing challenges. It is expected that future work would mainly focus on real‐time synchronous acquisition and discrimination of various kinds of information by combining with appropriate algorithms such as machine learning, and improve the accuracies of early pathological signal measurements from multiple perspectives.

## 3 Conclusion

The bio‐inspired ZIF‐L@Ti_3_CNT*_x_* composite was successfully fabricated to simultaneously mimic both the structure and ion conduction signal transformation mode of the synapse. Benefiting from the high proton conductivity capacity, fast ion transmission dynamics, long‐term stability, and excellent universality of ZIF‐L@Ti_3_CNT*_x_* composite, it was applied to build mutually non‐overlapping gas‐strain dual‐modal sensors. The ionic conduction working mechanism of the proposed sensor was first confirmed by an enhanced DMA gas response and protonic transmission dynamics under high‐humidity conditions, as well as by DFT simulation. Benefiting from the exquisite synaptic structure and superior ionic conduction mechanism, the bio‐inspired ZIF‐L@Ti_3_CNT*_x_* sensors could independently perceive DMA gas and stretching‐triggered strain with non‐overlapping electrical signals. In particular, it showed superiorities in DMA gas sensing at RT with a high response (*R*_a_ */R*_g_ = 420 ± 36 to 400 ppm), fast response/recovery speed (36/170 s), long‐term stability, and excellent compatibility with different substrates. Simultaneously, a high sensitivity to wide ranges of strain stretching (5–160%) with high speed (0.8/1.5 s for response and recovery times) and excellent cyclic stability (400 times) was also obtained. Due to the dual‐mode sensing properties and their non‐overlapping resistance variations, the proposed sensor was further integrated into a flexible smart terminal for wearable health monitoring application. The developed intelligent system could not only recognize expiratory DMA gas but also discriminate the physiological motion signals related with Parkinson. This work demonstrated a realistic prospect to establish bionic intelligent electronics for burgeoning telemedicine diagnosis.

## Conflict of Interest

The authors declare no conflict of interest.

## Supporting information

Supporting Information

Supplemental Movie1

Supplemental Movie2

Supplemental Movie3

Supplemental Movie4

## Data Availability

The data that support the findings of this study are available from the corresponding author upon reasonable request.
