# Supplementary material for: A Wearable Healthcare Platform Integrated with Biomimetical Ions Conducted Metal–Organic Framework Composites for Gas and Strain Sensing in Non‐Overlapping Mode
Source: Adv Sci (Weinh). 2023 Apr 20;10(18):2207663. doi: 10.1002/advs.202207663 (PMC10288278; doi:10.1002/advs.202207663)
Supplement: Supplementary file 1 — Supporting Information [file ADVS-10-2207663-s003.pdf]

ADVANCED SCIENCE
Open Access

Supporting Information

for *Adv. Sci.*, DOI 10.1002/advs.202207663

A Wearable Healthcare Platform Integrated with Biomimetical Ions Conducted Metal–Organic Framework Composites for Gas and Strain Sensing in Non-Overlapping Mode

Qingqing Zhou, Zixun Geng, Long Yang, Bo Shen, Zitong Kan, Yu Qi, Songtao Hu, Biao Dong, Xue Bai, Lin Xu, Hongwei Song* and Luquan Ren*

A Wearable Healthcare Platform Integrated with Biomimetically Ions Conducted Metal-Organic Framework Composites for Gas and Strain Sensing in Nonoverlapping Mode

Qingqing Zhou[a], Zixun Geng[a], Long Yang[a], Bo Shen[a], Zitong Kan[a], Yu Qi[a], Songtao Hu[a], Biao Dong[a], Xue Bai[a], Lin Xu[a, b]✉, Hongwei Song[a]✉, Luquan Ren[b]

[a]State Key Laboratory of Integrated Optoelectronics, College of Electronic Science and Engineering, Jilin University, Changchun, 130012, People's Republic of China.
[b]Key Laboratory of Bionic Engineering, Ministry of Education, College of Biological and Agricultural Engineering, Jilin University, Changchun 130025, People's Republic of China.

E-mail addresses: linxu@jlu.edu.cn (L, Xu); songhw@jlu.edu.cn (HW, Song)

Contents of the Supplementary Information

1. Experimental captions

1.1. Chemicals

1.2. Synthesis of the Ti_3CNT_x nanosheets

1.3. Modification of Ti_3CNT_x MXene

1.4. Preparation of the hierarchical ZIF-L@Ti_3CNT_x composites

1.5. Synthesis of flexible TPU nanofibers substrate

1.6. Characterizations of various ZIF-L@Ti_3CNT_x composites

1.7. Fabrication and measurement of various vapor sensors

1.8. Proton Conductivity Evaluation

1.9. Preparation of the flexible strain Sensor

1.10. Computational Details

1.11. Development of flexible intelligent wearable systems

1.12. Informed Consent

2. Figure and table captions

Figure S1. SEM images of the multilayered Ti_3CNT_x nanosheets.

Figure S2. (a) The AFM image and (b) film thickness profile of Ti_3CNT_x nanosheets.

Figure S3. A partial magnification of (002) diffraction peak of Ti_3CNT_x nanosheets and ZIF-L@Ti_3CNT_x composites.

Figure S4. The resistance variation curves of the Ti_3CNT_x, ZIF-L@Ti_3CNT_x and ZIF-L sensors after injecting 80 ppm DMA gas.

Figure S5. SEM images of the ZIF-$L_{1/2}$@Ti_3CNT_x, ZIF-$L_{1/8}$@Ti_3CNT_x and ZIF-$L_{1/16}$@Ti_3CNT_x composites.

Figure S6. The linear relationship of various sensors in the methylamine (DMA) vapor concentration in the range of 80-400 ppm at RT (including the ZIF-$L_{1/2}$@Ti_3CNT_x, ZIF-$L_{1/4}$@Ti_3CNT_x, ZIF-$L_{1/8}$@Ti_3CNT_x and ZIF-$L_{1/16}$@Ti_3CNT_x, respectively).

Figure S7. The response time and recovery time of the Ti_3CNT_x, ZIF-L@Ti_3CNT_x and ZIF-L sensors.

Figure S8. Various response values with error bar of the ZIF-L@Ti_3CNT_x sensor based on different electrodes (including the ceramic tube electrodes, ceramic planer interdigital electrodes, PI substrate with Au electrodes, butter paper and TPU substrates with Cu electrodes) to 55 ppm DMA gas.

Figure S9. The responses of the ZIF-L@Ti_3CNT_x sensors based on TPU substrate with Cu electrodes which are bended at different angles (0°, 30°, 60°, 90°, 120°, respectively) to 55 ppm DMA gas.

Figure S10. The responses of the Ti_3CNT_x, ZIF-L@Ti_3CNT_x and ZIF-L sensors to 80 ppm of the dry DMA gas.

Figure S11. The responses of the Ti_3CNT_x and ZIF-L sensors to 80 ppm of the DMA gas at varying humidity conditions from 30% to 90% RH.

Figure S12. (a-c) The calibration curves of Ti_3CNT_x, ZIF-L and ZIF-L@Ti_3CNT_x sensors under various RH (30–90%) with (as marked by solid lines) and without 80 ppm DMA gas (as marked by dotted lines), respectively.

Figure S13. Nyquist plots of the ZIF-L (a and b) and Ti_3CNT_x (c) sensing materials at 303k under 50% RH and 99% RH; (b) is the magnification of (a).

Figure S14. Nyquist plots of the ZIF-L particles to 100 ppm DMA at 303-343 K under 99% RH.

Figure S15. The structural configurations of ZIF-L placed with 1 water molecule.

Figure S16. DFT calculations of the proton-binding sites during relaxation of ZIF-L structure with 1 water molecule, the proton is highlighted by the green circle.

Figure S17. (a)The XPS profile of the O 1s orbits in Ti_3CNT_x and Ti_3CNT_{OH} samples; (b) The response curves of the ZIF-L@Ti_3CNT_x and ZIF-L@Ti_3CNT_{OH} sensors to 100 ppm DMA gas.

Figure S18. A dramatically different resistance variation trend of the ZIF-L@Ti_3CNT_x sensor to 20% of tensile strain (increase in resistance) and 80 ppm DMA gas stimuli (decrease in resistance).

Figure S19. The resistance variation of the ZIF-L@Ti_3CNT_x sensor when simultaneously exerted 1 ppm DMA gas and 40% strain deformation.

Figure S20. Dynamic response curves of the ZIF-L@Ti_3CNT_x strain sensor with

various stretching variables (5%, 10% and 15%).

Figure S21 Dynamic response curves of the Ti_3CNT_x (left) and ZIF-L@Ti_3CNT_x (right) strain sensors with 40% stretching deformation.

Figure S22. The response curves of the ZIF-L@Ti_3CNT_x and ZIF-L@Ti_3CNT_{OH} sensors to 40% strain.

Figure S23. The real-time response curves of a wearable smart health-care monitor integrated with a dual-mode flexible sensor to the simulated expiration (containing 1 ppm DMA gas).

Figure S24. The real-time response curves of the laryngeal (a and b) and facial irritation (c).

Figure S25. The real-time response curves from the movement of the hand joints, such as grabbing a cup/lid/beverage bottle (a-c), splaying (d and e) and bending fingers (f and g).

Figure S26. The real-time response curves from the movement of the knee joints, such as bending knees (a), walking (b) as well as sitting and bending knees (c).

Figure S27. Monitoring simulated expiration (a) and bending knees state (b) in real time, where the data dots in the yellow box represent abnormal response values.

Figure S28. The response of the flexible intelligent wearable system after bending the flexible circuit board for 50 times to 240 ppm DMA gas (a) and 20% strain (b).

Table S1. The binding energy (BE) and proportion of the Ti element in the Ti_3CNT_x, and ZIF-L @Ti_3CNT_x composites sensing materials.

Table S2. A comparison of the sensing properties of recently reported MXene and ZIF-based sensors with the as-prepared ZIF-L@Ti_3CNT_x sensor in this work.

Table S3. The proton conductivities of three sensors at different humidity.

Table S4. The proton conductivities of ZIF-L@Ti_3CNT_x and ZIF-L at different temperature.

Table S5. The comparison of sensing performance results of recently reported strain sensors with the ZIF-L@Ti_3CNT_x strain sensor in this work.

1. Experimental

1.1. Chemicals

Zinc nitrate hexahydrate ($Zn(NO_3)_2 \cdot 6H_2O$, AR) and *N*, *N*-dimethylformamide (DMF, 99.5%) were provided by Sinopharm Group (China). Lithium fluoride (LiF, AR) and 2-methylimidazole (2-MeIM, 98%) were obtained from Shanghai Macklin Biochemical Technology Co., Ltd (China). TI_3AlCN chunk was supplied by the Yiyi Technology Co., LTD. Hydrochloric acid (HCl, AR), Formaldehyde (HCHO, AR) and ammonia solution ($NH_3 \cdot H_2O$, 25%) were respectively acquired from Beijing Chemical Plant of China and Tianjin Fengchuan Chemical Reagent Technology Co., LTD. Methylamine (MA, 25 wt%), dimethylamine (DMA, 40 wt%) and Trimethylamine water solution (TMA, 30 wt%) were all purchased from Tianjin Beilian Fine Chemicals Development Co. LTD. Standard NH_3 gas for the sensing measurement were obtained from Dalian Special Gases Co., Ltd (China). No further purification was performed for all chemical reagents and the deionized (DI) water was utilized in the whole experimental process.

1.2. Synthesis of the Ti_3CNT_x nanosheets

To chemically exfoliate the bulk Ti_3AlCN, 2 g of LiF powder was firstly immersed into 20 mL of HCl (9 M) and stirred for 15 min at 35°C in a water bath. Then, 1 g of Ti_3AlCN chunk was slowly added into the above solution and kept stirring for 24 h. After the etching procedure, HCl and LiF residuals in the supernatant were removed and the precipitates were purified and washed with deionized (DI) water by repetitive centrifugation (3500 rpm for 10 mins) until the pH value of the solution approached to 6. To obtain monolayer Ti_3CNT_x nanosheets, the above-washed precipitates were immersed in 200 mL of DI water and sonicated for 1 h at 18°C. Finally, after centrifugation (3500 rpm) for 30 mins, the upper suspension was collected as the delaminated Ti_3CNT_x nanosheets and the resultant sediment was collected as multilayered Ti_3CNT_x nanosheets.

1.3. Modification of Ti_3CNT_x MXene

Firstly, LiOH powder (45 wt%) was dispersed in the delaminated Ti_3CNT_x (30ml, 2.5 mg/mL) solution and stirred for 6 h at room temperature. Then, the mixture was collected after washed repeatedly with DI water by centrifugation to obtain Ti_3CNT_{OH} nanosheets.

1.4. Preparation of the hierarchical ZIF-L@Ti_3CNT_x composites

For *in-situ* constructing the ZIF-L@Ti_3CNT_x composites, $Zn(NO_3)_2{\cdot}6H_2O$ and 2-MeIM (molar ratio, 1:2, 1:4, 1:8 and 1:16, respectively) were separately dissolved into the delaminated Ti_3CNT_x suspension (2 mg/mL, 15 mL) and sonicated at room temperature (RT) for 20 mins to ensure a robust assembly of ZIF-L onto the MXene nanosheet. Then, the obtained MOF@MXene composites were dried and collected for subsequent gas/strain sensing tests. For clarity, the corresponding samples synthesized by diverse mole ratios (1:2, 1:4, 1:8 and 1:16) are defined as ZIF-$L_{1/2}$@Ti_3CNT_x, ZIF-$L_{1/4}$@Ti_3CNT_x, ZIF-$L_{1/8}$@Ti_3CNT_x and ZIF-$L_{1/16}$@Ti_3CNT_x, respectively. Noticeably, the ZIF-$L_{1/4}$@Ti_3CNT_x composites has the optimal sensing performance after a series of gas sensing tests, so it is mainly discussed and further abbreviated as ZIF-L@Ti_3CNT_x composites. In comparison, similar experimental treatment were utilized to fabricate the pristine flower-like ZIF-L particles by dissolving $Zn(NO_3)_2{\cdot}6H_2O$ and 2-MeIM (mole ratio was1:4) into 15 mL of DI water without the delaminated Ti_3CNT_x nanosheets. In addition, the ZIF-L@Ti_3CNT_{OH} was fabricated by the procedure similar to that of ZIF-L@Ti_3CNT_x, except for changing Ti_3CNT_x to Ti_3CNT_{OH}.

1.5. Synthesis of flexible TPU nanofibers substrate

Firstly, the electrospinning precursor solution was fabricated by dissolving 6.0 g of TPU powder in 20 mL of DMF/THF (3:1, v/v) solvent, followed by stirring for 5 h at RT. Subsequently, single-spinneret electrospinning was performed at a DC voltage of 15 kV, a receiving distance of 15cm and the feed rate of 20 μL min^{-1}, respectively.

1.6. Characterizations of various ZIF-L@Ti_3CNT_x composites

The biomimetic synaptic architecture of ZIF-L@Ti_3CNT_x composites were confirmed by field-emission scanning electron microscope (SEM) operating with an

accelerating voltage of 15 kV (FESEM, JEOL JSM-7500F, Japan). Transmission electron microscopy (TEM) and high-resolution TEM (HRTEM) images were collected from a JEM-2010 transmission electron microscope (JEOL, Japan) operating with a working voltage of 200 kV, which coupled with an energy dispersive X-ray spectrometer (EDX). The thickness of MXene nanosheets was determined by the atomic force microscopy (AFM, Bruker Dimension Icon). The structure of the as-prepared MXene nanosheets and ZIF-L@Ti_3CNT_x composites were characterized by a RigakuTTR III X-ray diffractometer (Tokyo, Japan) with a monochromatised Cu target radiation source (λ= 1.5406 Å). Fourier transform infrared spectroscopy (FTIR) spectra were recorded on a Vertex 80 V (Bruker) FTIR spectrometer using a KBr pellet in the range of 400-4000 cm^{-1}. The thermal decomposition behavior of samples was observed by thermogravimetric analysis (TGA, Q500, TA) and differential thermogravimetry analysis (DTG). The X-ray photoelectron spectra (XPS) was obtained on a Thermo Scientific ESCALAB 250 apparatus (Thermo Fisher Scientific, Waltham, MA, USA). Electrochemical impedance spectroscopy (EIS) tests were recorded on a PMC 500/LO multichannel workstation in the frequency range 0.1–1 MHz at AC amplitude of 10 mV.

1.7. Fabrication and measurement of various vapor sensors

To fabricate the gas sensors, 60 μL of fresh Ti_3CNT_x, ZIF-L and ZIF-L@Ti_3CNT_x composites solution (5 mg/mL) were directly dripped onto the rigid ceramic tube and alumina flat-based substrates, or onto flexible commercial polyimide (PI), as-spinning TPU and transparent sulfate paper-based substrates. Herein, different types of substrates are used to illustrate the superior compatibility of the prepared sensing materials which can be flexibly applied to various practical scenarios. The chemiresistive response value of all sensors is defined as R_a/R_g, where R_a and R_g are the steady-state resistance of sensors in air and in analyzed gases, respectively. The response / recovery time are refined as the time duration from the initial resistance value to 90% of the final equilibrium state. The gas sensing properties were assessed by WS-30 sensing instrument (Weisheng Instruments Co., Zhengzhou, China) equipped with a 30-channel multiplexer. Most chemiresistive

response measurements of the sensing layer were performed at RT which varied in the range of 25–30°C. The used standard analyte vapors including MA, DMA, TMA, HCHO, DMF, $NH_3{\cdot}H_2O$ were fabricated by a static volumetric method. Specifically, a certain amount of analyte solutions was injected into an evaporator placed in an 18 L gas sensing test chamber by using a microsyringe. The vapor concentrations were calculated by the equation (1):[1]

$$C=\frac{22.4\ dPV_S}{MV}\text{x } 10^{-9} * \frac{273+T_r}{273+T_b} \quad (1)$$

Where C is the vapor concentration (ppm), d, p and M are the density (g/cm^3), purity and relative molecular mass (g/mol) of the solution liquid, V and V_s are the volume of gas sensing test chamber (L) and injected liquid sample (mL), T_r and T_b (°C) are the temperature of surrounding and sensing chamber, respectively.

1.8. Proton Conductivity Evaluation

Before measurement, the ZIF-L@Ti_3CNT_x solution was dropped to a finger electrode (1×1 cm^2). The proton conductivity was determined by equation (2):[2]

$$\sigma = \frac{1}{R_f} * \frac{d}{l\ (N-1)t} \quad (2)$$

where R_f is the resistance (Ω), d is the space between the electrode teeth (50 μm), l is the length of the teeth (7000 μm), N is the number of electrodes (20), t is the thickness of the samples (1-2 μm).

The activation energy (E_a) was calculated by equation (3):

$$(\sigma) = \sigma_0 \exp\left(-\frac{E_a}{k_B T}\right) \quad (3)$$

where σ_0 is the pre-exponential factor, k_B is the Boltzmann constant, T is the temperature in Kelvin.

1.9. Preparation of the flexible strain Sensor

The ZIF-L@Ti_3CNT_x solution was filtrated onto the electrospun TPU membrane, and then, Cu tape electrode was adhered to both side of the TPU substrate to construct the flexible strain sensor, where length, width and thickness of the substrate is 2, 0.5 and 0.13 mm, respectively.

1.10. Computational Details

Density functional theory (DFT) calculations were performed using the Vienna Ab

initio Simulation Package (VASP) based on the pseudopotential plane wave (PPW) method.[3] The perdew-Bueke-Ernzerhof (PBE) functional was used to describe exchange-correlation effects of electrons.[4] The projected augmented wave (PAW) potentials were chosen to describe the ionic cores and took valence electrons into account using a plane wave basis set with a kinetic energy cutoff of 500 eV.[5] In order to simulate the proton transfer under humid and dry environment, hydrogen network built by water molecules and imidazole molecules were modelled. The DMA/ZIF-L system is placed with 12 water molecules to simulate high humidity environment, and with 1 water molecules is to simulate the dry environment. All structures were first optimized to reach their most stable configuration. During the geometry optimizations, all the atom positions were allowed to relax. In this work, the Brillouin-zone sampling were conducted using Monkhorst-Pack (MP) grids of special points with the separation of 0.04 $Å^{-1}$.[6] The convergence criterion for the electronic self-consistent field (SCF) loop was set to 1×10^{-5} eV/atom. The atomic structures were optimized until the residual forces were below 0.05 $eVÅ^{-1}$. The barrier of proton transport within hydrogen network was calculated by CI-NEB method. [7]

1.11. Development of flexible intelligent wearable systems

To be specific, the ESP32 chip is performed in this device, which is integrated with Wi-Fi connectivity developed by Espressif Systems (Shanghai) Co., Ltd. In addition, an integrated electric circuit (ESP32 module) is designed to read the resistance data of the flexible sensor and upload it to the cloud by using a divider circuit structure, followed by a conversion to digital domain via an analog to digital converter (ADC). The cloud uses Alibaba Cloud IoT, a cloud computing brand under Alibaba Group, which provides reliable device connectivity and communication capabilities. The cloud will save the analyzable data collected from the flexible sensors in the server database and forward it to the smartphones or computers terminal. The mobile terminal uses a self-developed supporting application to receive and process the forwarded data from the cloud. To meet the human-centered design requirements for wearable monitoring, the flexible printed circuit board (FPC) is integrated with an individual channels to monitor sensing signals. The flexible sensor

is soldered to the FPC and connected to the pins by individually drawing out copper wires at both ends. One channel of the FPC board was utilized to the acquisition of human physiological signals. Firstly, the sensor is applied to identify the simulated expiratory signals.

1.12. Informed Consent

All procedures of the investigation in human subjects were performed in compliance with relevant laws and institutional guidelines of Jilin University. Prior to participation in the experiments, informed consent was obtained for all experimentation from human subjects. The privacy rights of human subjects have always been observed.

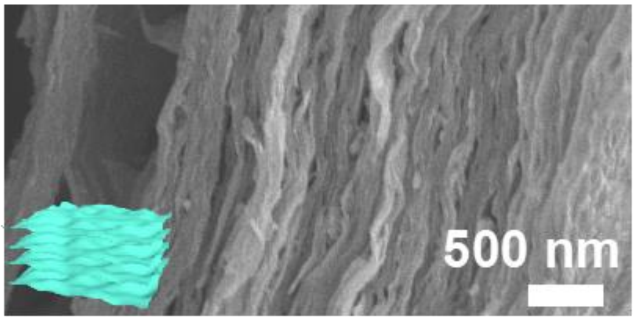

Figure S1. SEM images of multilayered Ti_3CNT_x nanosheets.

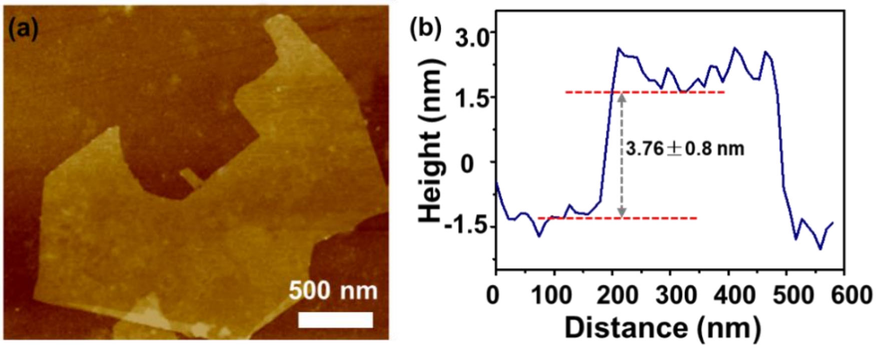

Figure S2. (a) The AFM image and (b) film thickness profile of Ti_3CNT_x nanosheets.

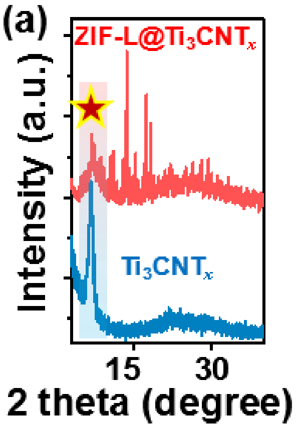

Figure S3. A partial magnification of (002) diffraction peak of Ti_3CNT_x nanosheets and ZIF-L@Ti_3CNT_x composites.

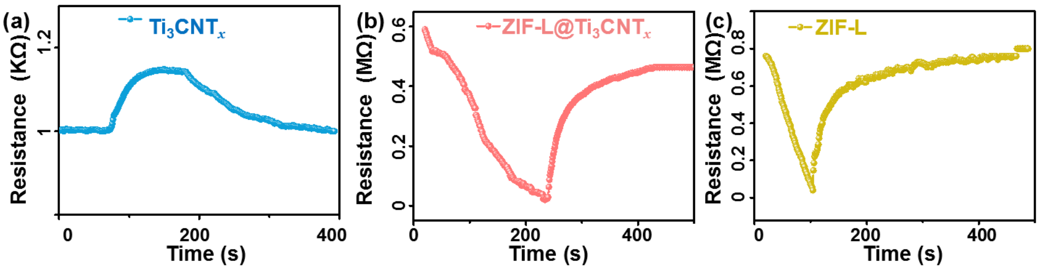

Figure S4. The resistance variation curves of the Ti_3CNT_x, ZIF-L@Ti_3CNT_x and ZIF-L sensors after injecting 80 ppm DMA gas.

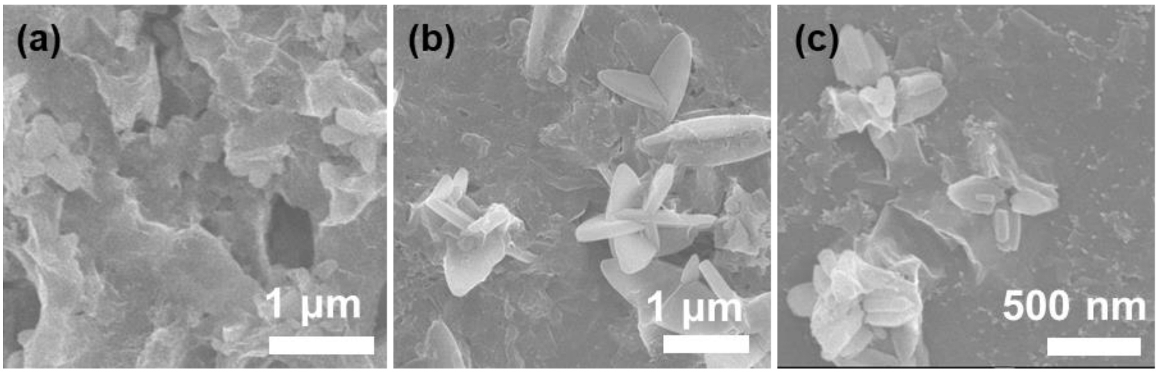

Figure S5. SEM images of the ZIF-$L_{1/2}$@Ti_3CNT_x, ZIF-$L_{1/8}$@Ti_3CNT_x and ZIF-$L_{1/16}$@Ti_3CNT_x composites.

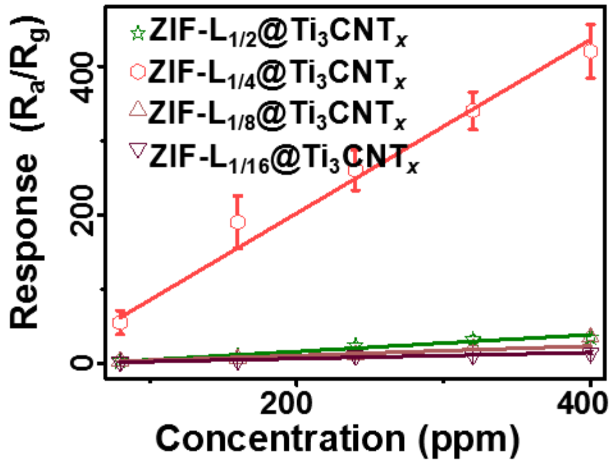

Figure S6. The linear relationship of various sensors in the methylamine (DMA) vapor concentration in the range of 80-400 ppm at RT (including the ZIF-$L_{1/2}$@Ti_3CNT_x, ZIF-$L_{1/4}$@Ti_3CNT_x, ZIF-$L_{1/8}$@Ti_3CNT_x and ZIF-$L_{1/16}$@Ti_3CNT_x, respectively).

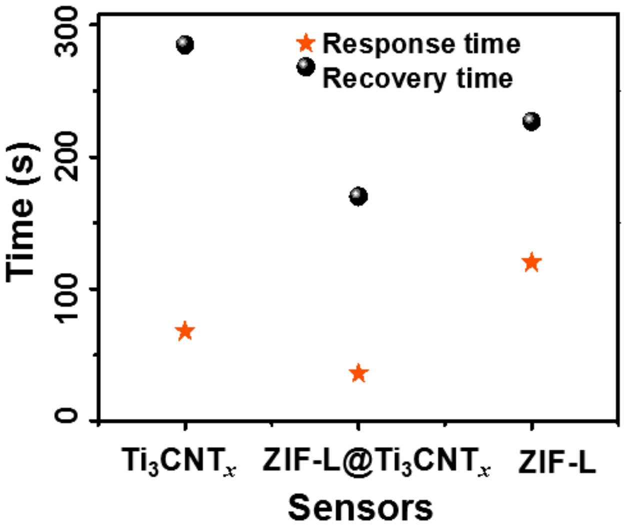

Figure S7. The response time and recovery time of the Ti_3CNT_x, ZIF-L@Ti_3CNT_x and ZIF-L sensors.

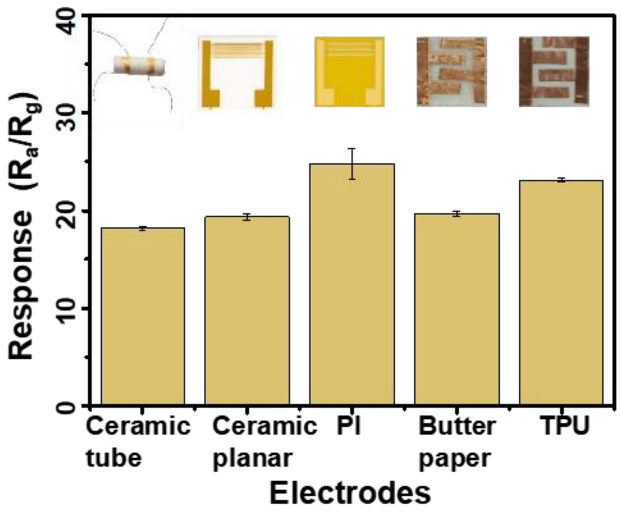

Figure S8. Various response values with error bar of the ZIF-L@Ti_3CNT_x sensor based on different electrodes (including the ceramic tube electrodes, ceramic planer interdigital electrodes, PI substrate with Au electrodes, butter paper and TPU substrates with Cu electrodes) to 55 ppm DMA gas.

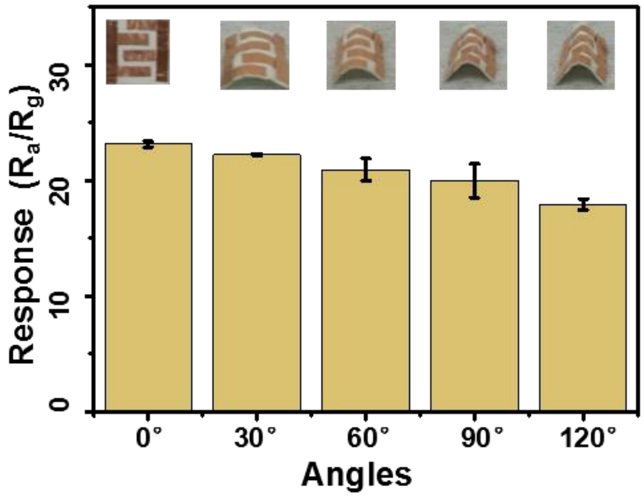

Figure S9. The responses of the ZIF-L@Ti_3CNT_x sensors based on TPU substrate with Cu electrodes which are bended at different angles (0°, 30°, 60°, 90°, 120°, respectively) to 55 ppm DMA gas.

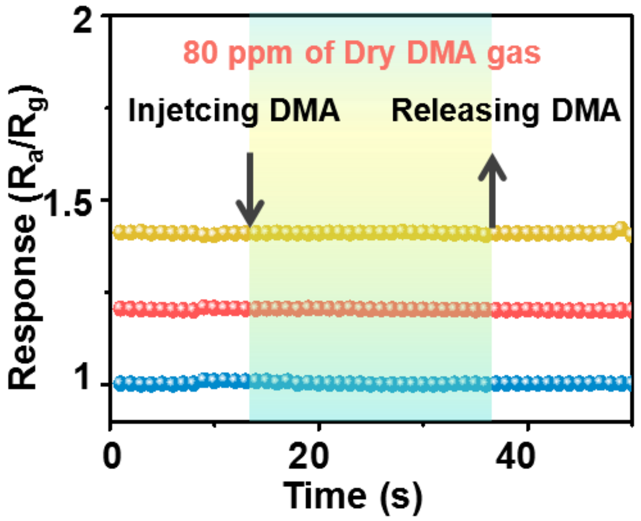

Figure S10. The responses of the Ti_3CNT_x, ZIF-L@Ti_3CNT_x and ZIF-L sensors to 80 ppm of the dry DMA gas.

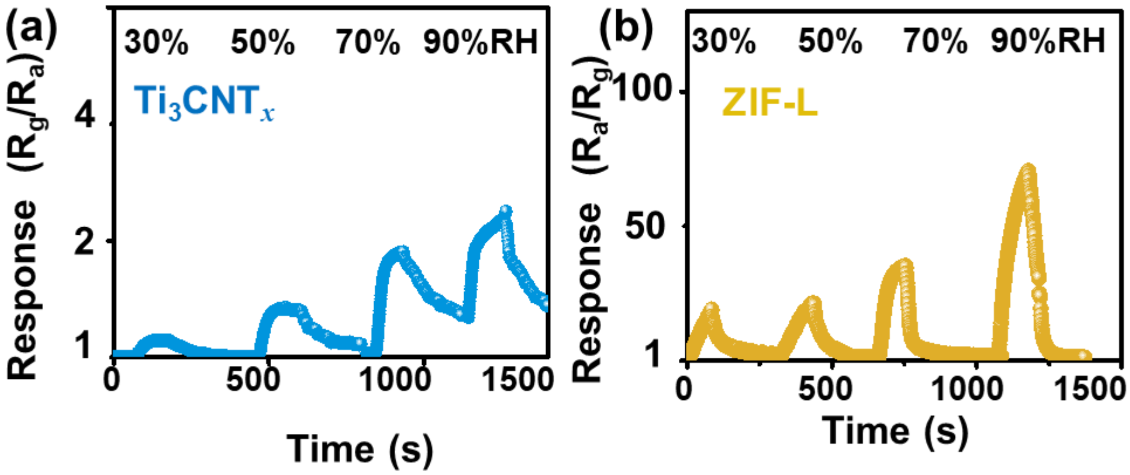

Figure S11. The responses of the Ti_3CNT_x and ZIF-L sensors to 80 ppm of the DMA gas at varying humidity conditions from 30% to 90% RH.

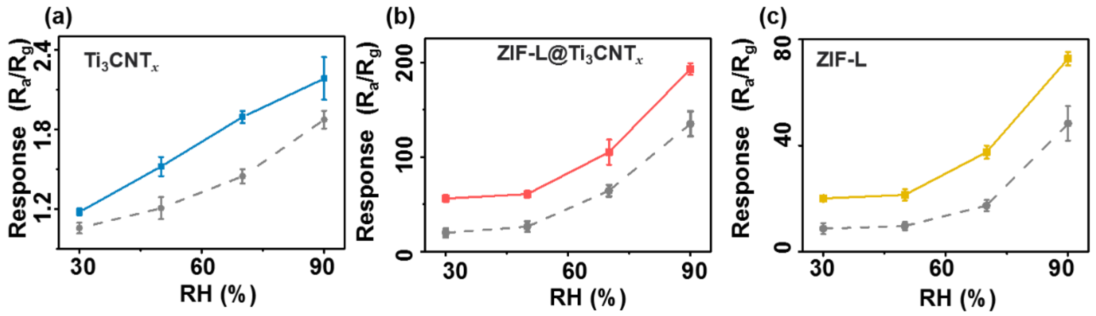

Figure S12. (a-c) The calibration curves of Ti_3CNT_x, ZIF-L and ZIF-L@Ti_3CNT_x sensors under various RH (30–90%) with (as marked by solid lines) and without 80 ppm DMA gas (as marked by dotted lines), respectively.

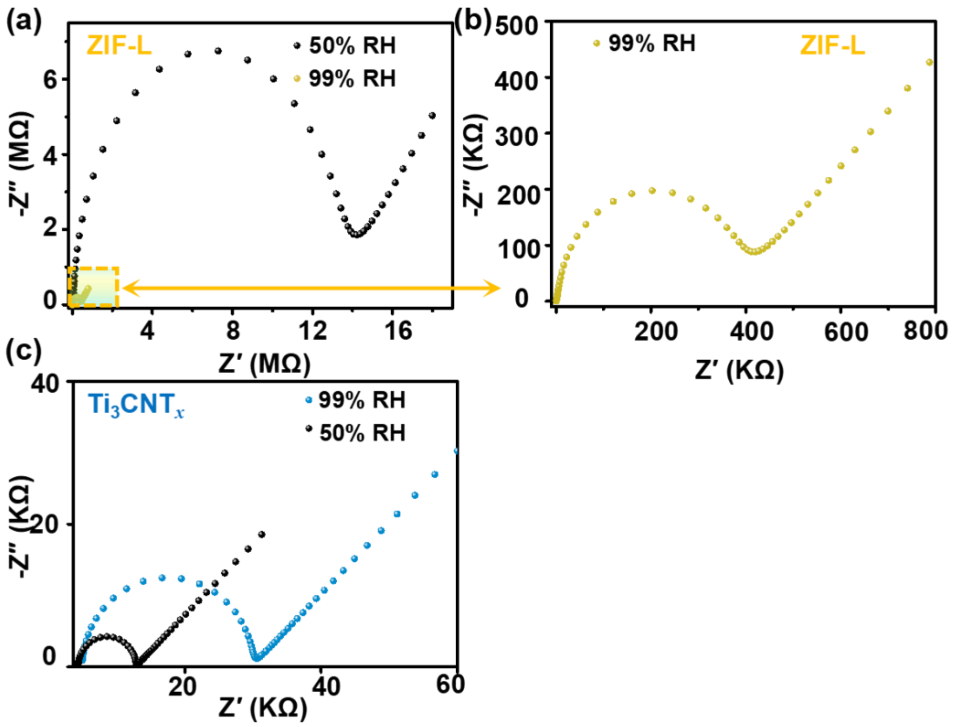

Figure S13. Nyquist plots of the ZIF-L (a and b) and Ti_3CNT_x (c) sensing materials at 303k under 50% RH and 99% RH; (b) is the magnification of (a).

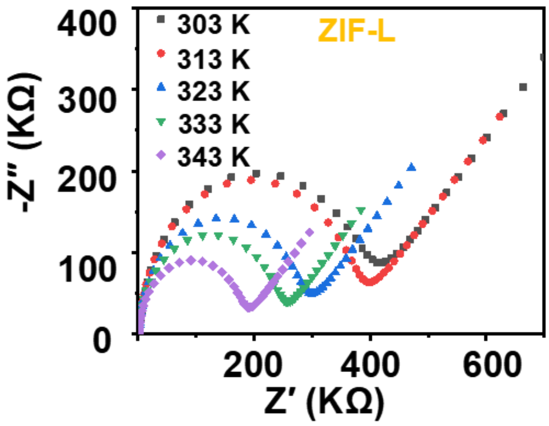

Figure S14. Nyquist plots of the ZIF-L particles to 100 ppm DMA at 303-343 K under 99% RH.

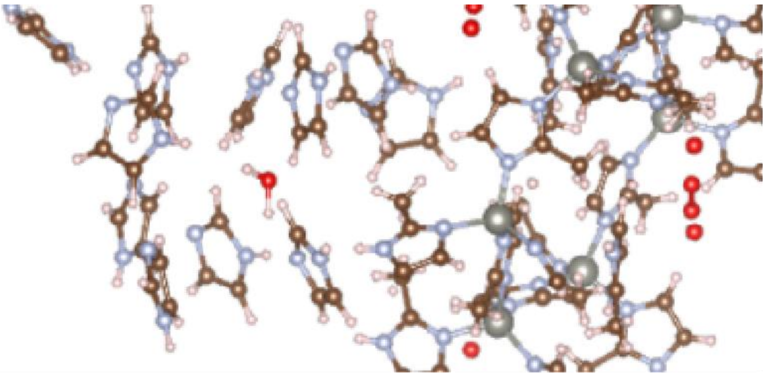

Figure S15. The structural configurations of ZIF-L placed with 1 water molecule.

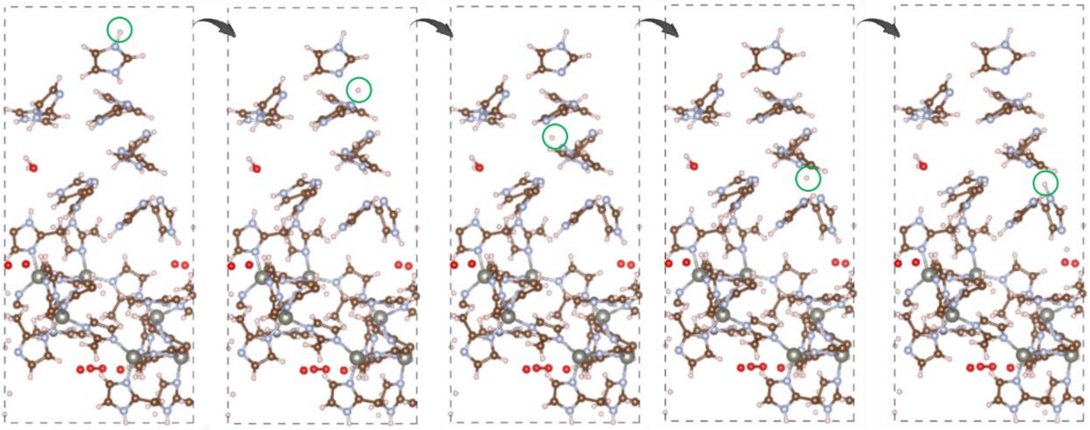

Figure S16. DFT calculations of the proton-binding sites during relaxation of ZIF-L structure with 1 water molecule, the proton is highlighted by the green circle.

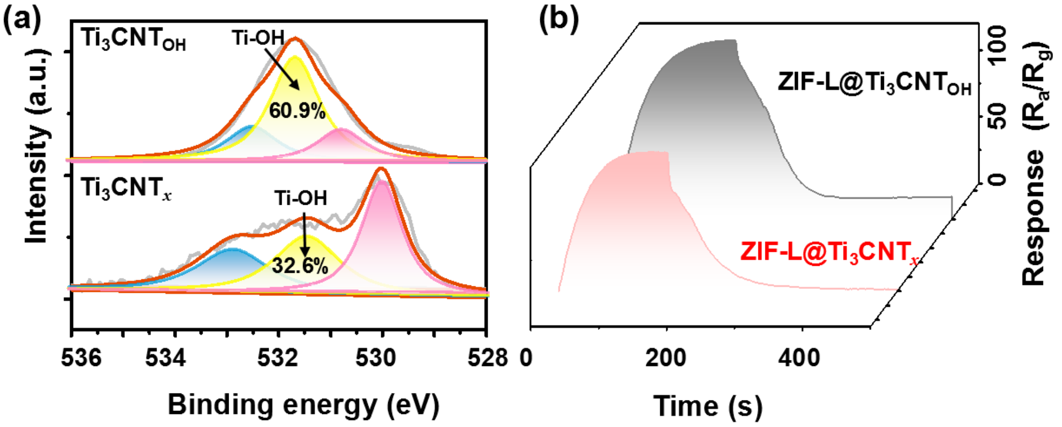

Figure S17. (a)The XPS profile of the O 1s peak in Ti_3CNT_x and Ti_3CNT_{OH} samples; (b) The response curves of the ZIF-L@Ti_3CNT_x and ZIF-L@Ti_3CNT_{OH} sensors to 100 ppm DMA gas.

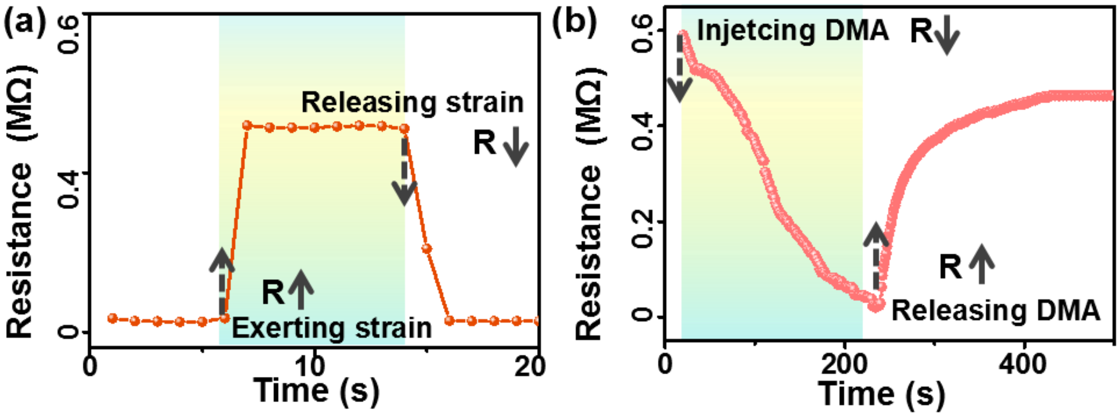

Figure S18. A dramatically different resistance variation trend of the ZIF-L@Ti_3CNT_x sensor to 20% of tensile strain (increase in resistance) and 80 ppm DMA gas stimuli (decrease in resistance).

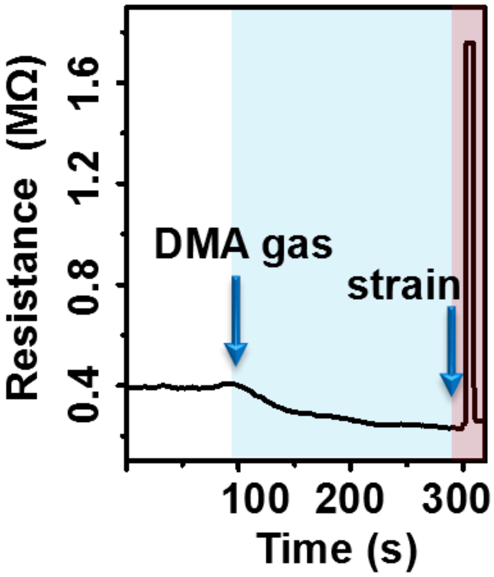

Figure S19. The resistance variation of the ZIF-L@Ti_3CNT_x sensor when simultaneously exerted 1 ppm DMA gas and 40% strain deformation.

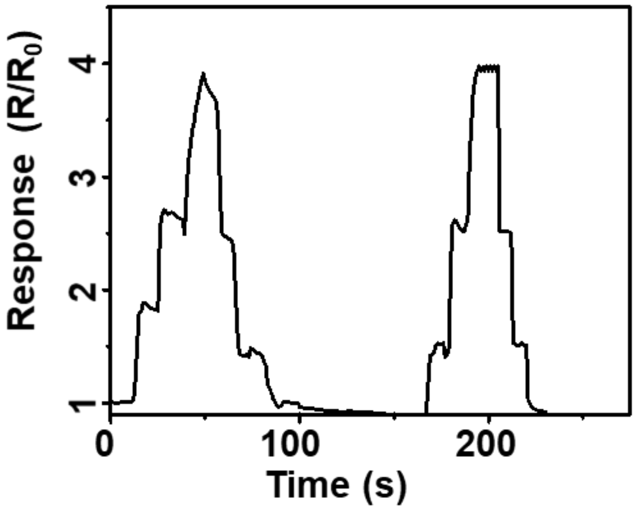

Figure S20. Dynamic response curves of the ZIF-L@Ti_3CNT_x strain sensor with various stretching variables (5%, 10% and 15%).

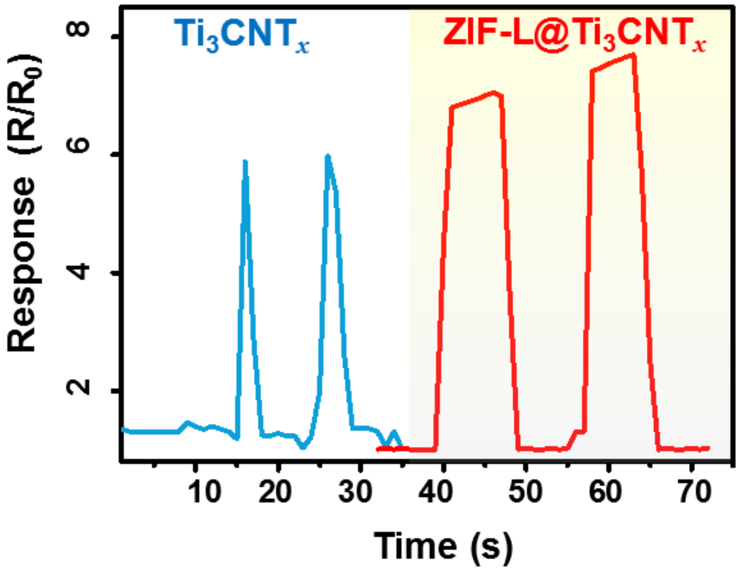

Figure S21. Dynamic response curves of the Ti_3CNT_x (left) and ZIF-L@Ti_3CNT_x (right) strain sensors with 40% stretching deformation.

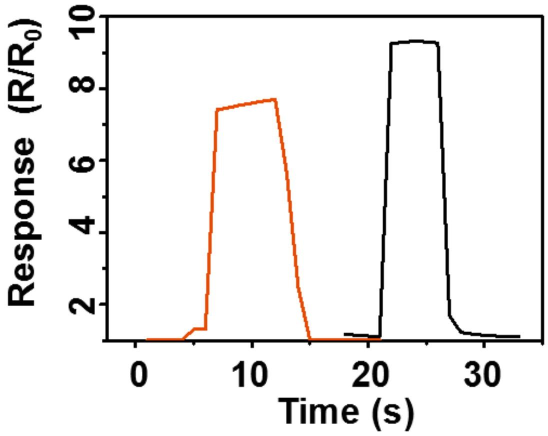

Figure S22. The response curves of the ZIF-L@Ti_3CNT_x and ZIF-L@Ti_3CNT_{OH} sensors to 40% strain.

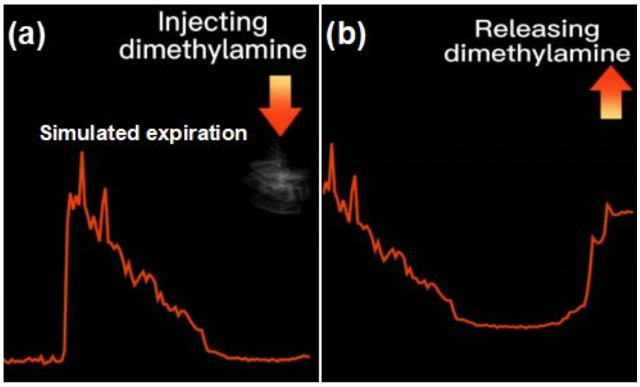

Figure S23. The real-time response curves of a wearable smart health-care monitor integrated with a dual-mode flexible sensor to the simulated expiration (containing 1 ppm DMA gas).

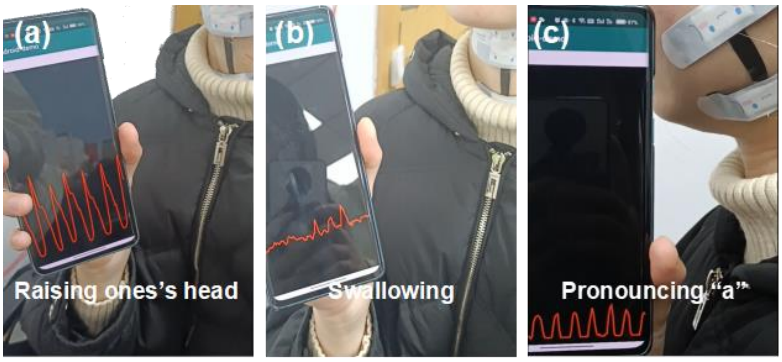

Figure S24. The real-time response curves of the laryngeal (a and b) and facial irritation (c).

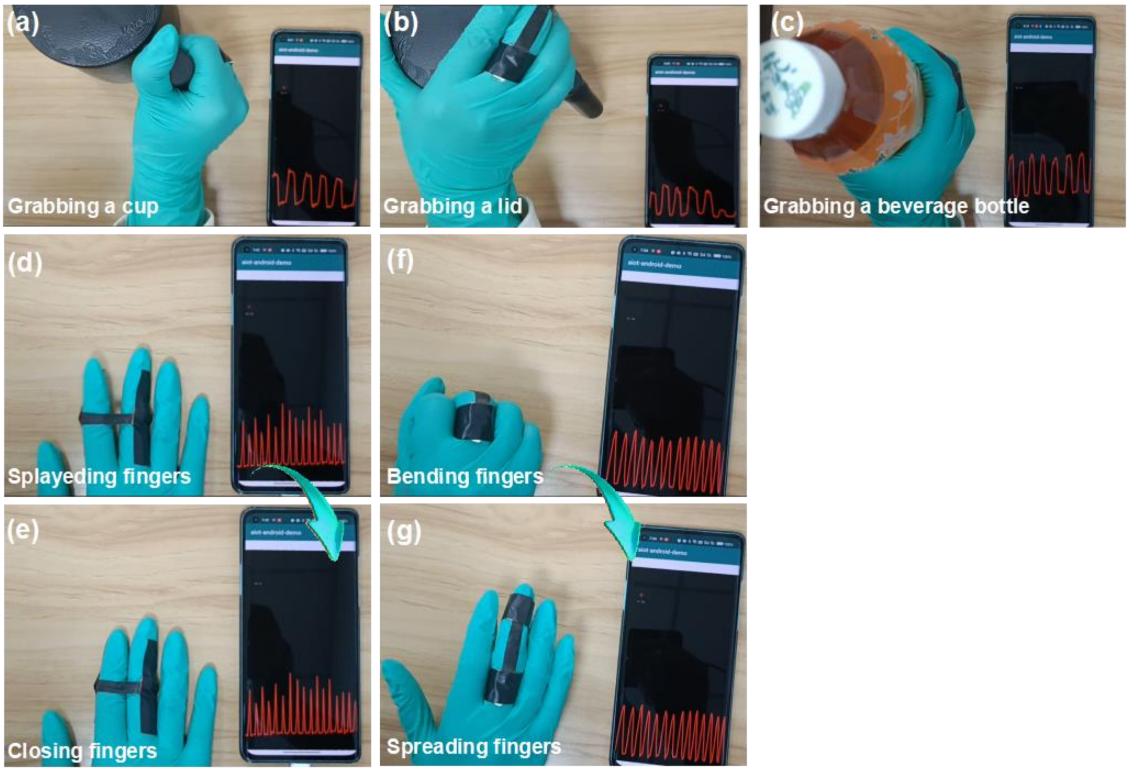

Figure S25. The real-time response curves from the movement of the hand joints, such as grabbing a cup/lid/beverage bottle (a-c), splaying (d and e) and bending fingers (f and g).

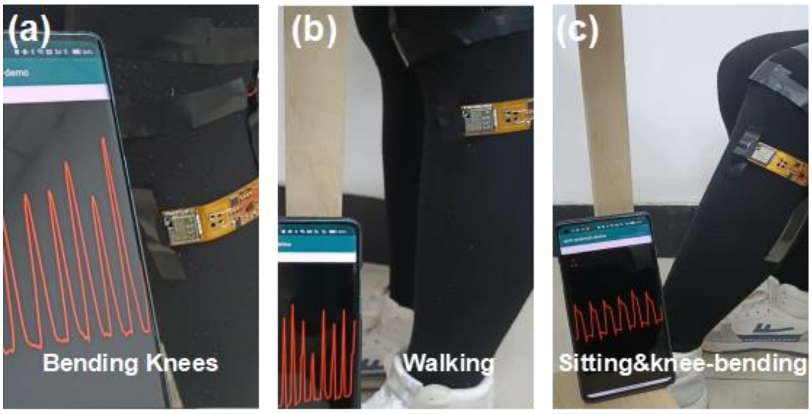

Figure S26. The real-time response curves from the movement of the knee joints, such as bending knees (a), walking (b) as well as sitting and bending knees (c).

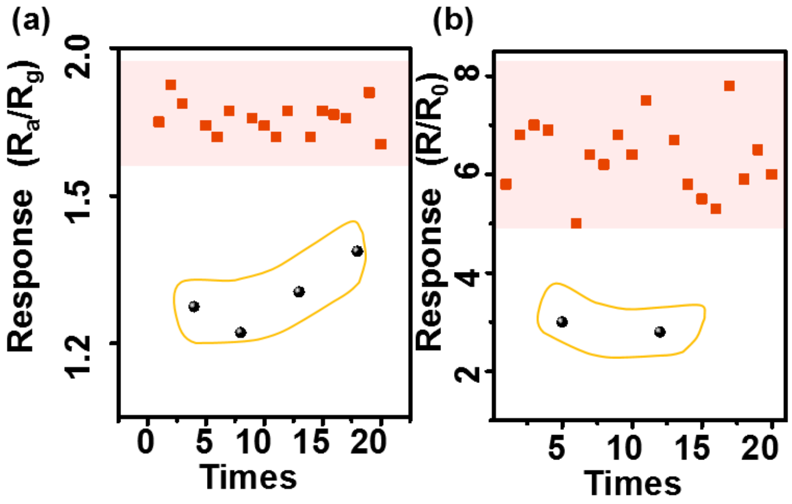

Figure S27. Monitoring simulated expiration (a) and bending knees state (b) in real time, where the data dots in the yellow box represent abnormal response values.

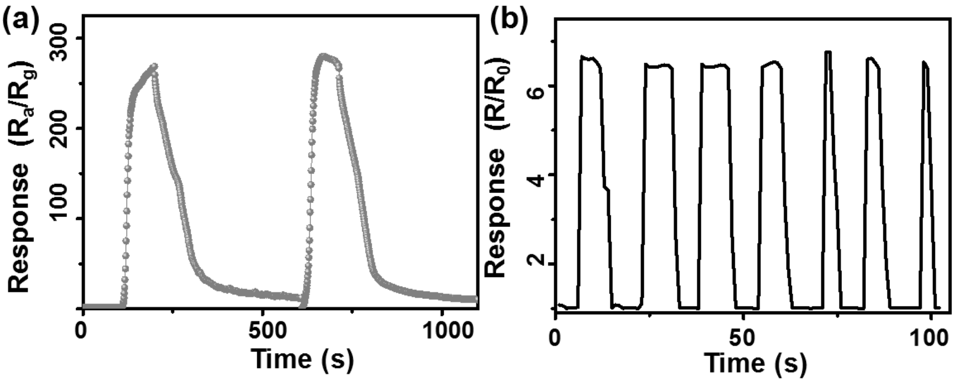

Figure S28. The response of the flexible intelligent wearable system after bending the flexible circuit board for 50 times to 240 ppm DMA gas (a) and 20% strain (b).

Table S1. The binding energy (BE) and proportion of the Ti element in the Ti_3CNT_x, and ZIF-L@Ti_3CNT_x composites sensing materials.

**Samples**	**BE (eV)/proportion**			
	**Ti-C**	**Ti-N**	**Ti-O**	**Ti-F**
Ti_3CNT_x	455.2(458.8)/ 24.83%	455.9(461.3)/ 27.56%	456.6(462.4)/ 20.48%	457.5(463.8)/ 27.17%
ZIF-L@Ti_3CNT_x composites	454.5(458.0)/ 28.32%	455.2(460.6)/ 27.36%	455.9(461.9)/ 19.34%	456.9(463.5)/ 24.97%

Table S2. A comparison of the sensing properties of recently reported MXene and ZIF-based sensors with the as-prepared ZIF-L@Ti_3CNT_x sensor in this work.

**Sensing Materials**	**Targets**	**Temp (°C)**	**[a] R_{res} / Conc. (R_a/R_g/ppm)**	**T_{res} / T_{rec} (s)**	**RH**	**Ref.**
$Ti_3C_2T_x$/SnO	NH_3	RT	9.96/200	61/119	28%	[8]
PANI-MXene	NH_3	RT	27/5	9/9	22%	[9]
MXene/SnO_2	NH_3	RT	40/50	36/44	45%	[10]
Pt SA-$Ti_3C_2T_x$ FET	TEA	RT	1.04/1	113/-	–	[11]
$Ti_3C_2T_x/V_2O_5/CuWO_4$	NH_3	RT	53.5/51	10/5	–	[12]
$Ti_3C_2T_x$ @TiO_2/MoS_2	NH_3	RT	4/100	117/88	75%	[13]
$Mo_2TiC_2T_x/MoS_2$	NO_2	RT	5.2/50	34.8/140.5	50%	[14]
$Ti_3C_2T_x/WO_3$	NH_3	RT	1.29/1	119/228	–	[15]
Fe_2O_3@ZnO@ZIF-8	H_2S	200	32.2/10	18.0/31.8	–	[16]
ZnO@ZIF-8	C_2H_5OH	160	35.9/100	23/31	–	[17]
ZnO@ZIF–8	HCHO	300	13/100	16/9	50-60 %	[18]
$CoSnO_3$@MOF@PDMS	H_2S	160	12/5	–	0	[19]
ZnO/Pd@ZIF-8	CH_4	80	1.04/1	3.5/4.8 min	–	[20]
ZIF-L@Ti_3CNT_x	DMA	RT	55/80	36/170	35	This work

[a] Where R_{res} is the response; R_a and R_g represent the resistance of the sensors to the air and the target gas, respectively; T_{res}/T_{rec} is the response/recovery times; Temp: Temperature; RT: Room temperature; Conc.: Concentration; RH: Relative Humidity; SA: single atoms; TEA: triethylamine; FET: field effect transistor; PDMS: polydimethylsiloxane.

Table S3. The proton conductivities of three sensors at different humidity.

**Samples**	**Proton conductivity (σ, ×10^{-6} S cm^{-1})**	
	**55% RH**	**99% RH**
Ti_3CNT_x	606.5	247.7
ZIF-L@Ti_3CNT_x	0.31	12
ZIF-L	0.29	10.0

Table S4. The proton conductivities of ZIF-L@Ti_3CNT_x and ZIF-L at different temperature.

**Samples**	**Proton conductivity (σ, ×10^{-6} S cm^{-1})**				
	**303 K**	**313 K**	**323 K**	**333 K**	**343 K**
ZIF-L@Ti_3CNT_x	12.1	15.8	18.8	23.4	29.7
ZIF-L	10.0	11.3	15.5	17.6	25.4

Table S5. The comparison of sensing performance results of recently reported strain sensors with the ZIF-L@Ti_3CNT_x strain sensor in this work.

Samples	Type of sensor	Stretchability (%)	Gauge factor	Linearity	Ref.
Ag NWs[a]/TPU/PDMS	Resistive	50	12.9	linearity	[21]
SWCNTs/MWCNTs/TPU	Capacitive	300	1.67	linearity	[22]
GNP/PU	Capacitive	30	3.5	linearity	[23]
CNTs/PDMS	Capacitive	150	1	linearity	[24]
graphene/carbon black yarn	Resistive	60	1.46- 5.62	linearity	[25]
Au/PDMS	Resistive	80	20 /350	Nonlinearity	[26]
B/CNTs/TPU	Resistive	3	6	linearity	[27]
SCNC-CNT/PDMS	Resistive	100	10.77/23.95/37.11	Three linear regions	[28]
CNT/CB	Resistive	80	7.747	linearity	[29]
CNTs-CB/PDMS	Resistive	300	0.91-13.1	Three linear regions	[30]
ZIF-L@Ti_3CNT_x /TPU	Resistive	160	12.23/37.23	Two linear regions	This work

[a] NWs: Nanowires; TPU: Thermoplastic polyurethane; PDMS: Polydimethylsiloxane; SWCNTs: Single-walled carbon nanotubes; MWCNTs: Multi-walled Carbon Nanotubes; GNPs: Graphite nanoplatelets; CB: Carbon black; SCNC: Silylated cellulose nanocrystal.

References

[1] K. Zhang, R. Hu, G. Fan, G. Li, *Sens. Actuators, B* **2017**, *243*, 721.

[2] H. Wu, M. Almalki, X. Xu, Y. Lei, F. Ming, A. Mallick, V. Roddatis, S. Lopatin, O. Shekhah, M. Eddaoudi, H. N. Alshareef, *J. Am. Chem. Soc.***2019**, *141*, 20037.

[3] a)G. K. A, J. F. b, *Comp Mater Sci* **1996**, *6*, 15; b)G. Kresse, J. Hafner, *Phys. Rev. B: Condens. Matter* **1994**, *49*, 14251.

[4] Perdew, Burke, Ernzerhof, *Phys. Rev. Lett.* **1996**, *77*, 3865.

[5] a)Blochl, Jepsen, Andersen, *Phys. Rev. B: Condens. Matter* **1994**, *49*, 16223; b)G. Kresse, D. Joubert, *Phys. Rev. B.* **1999**, *59*, 1758.

[6] H. J. Monkhorst, J. D. Pack, *Phys. Rev. B.* **1976**, *13*, 5188.

[7] a)G. Henkelman, H. Jonsson, *J. Chem. Phys.* **2000**, *113*, 9978; b)G. Henkelman, B. P. Uberuaga, H. Jonsson, *J. Chem. Phys.* **2000**, *113*, 9901.

[8] L. Yao, X. Tian, X. Cui, R. Zhao, M. Xiao, B. Wang, X. Xiao, Y. Wang, *Sens. Actuators, B* **2022**, *358*, 131501.

[9] X. Wang, D. Zhang, H. Zhang, L. Gong, Y. Yang, W. Zhao, S. Yu, Y. Yin, D. Sun, *Nano Energy* **2021**, *88*, 106242.

[10] T. He, W. Liu, T. Lv, M. Ma, Z. Liu, A. Vasiliev, X. Li, *Sens. Actuators, B* **2021**, *329*, 129275.

[11] B. Zong, Q. Xu, S. Mao, *ACS Sensors* **2022**, *7*, 1874.

[12] F. Ranjbar, S. Hajati, M. Ghaedi, K. Dashtian, H. Naderi, J. Toth, *J. Hazard. Mater.* **2021**, *416*, 126196.

[13] X. Tian, L. Yao, X. Cui, R. Zhao, T. Chen, X. Xiao, Y. Wang, *J. Mater. Chem. A* **2022**, *10*, 5505.

[14] Q. Zhao, W. Zhou, M. Zhang, Y. Wang, Z. Duan, C. Tan, B. Liu, F. Ouyang, Z. Yuan, H. Tai, Y. Jiang, *Adv. Funct. Mater.* **2022**, *32*, 2203528.

[15] X. Guo, Y. Ding, D. Kuang, Z. Wu, X. Sun, B. Du, C. Liang, Y. Wu, W. Qu, L. Xiong, Y. He, *J. Colloid. Interf. Sci.* **2021**, *595*, 6.

[16] L.-Y. Zhu, X.-Y. Miao, L.-X. Ou, L.-W. Mao, K. Yuan, S. Sun, A. Devi, H.-L. Lu, *Small* **2022**, 2204828.

[17] G. Ren, Z. Li, W. Yang, M. Faheem, J. Xing, X. Zou, Q. Pan, G. Zhu, Y. Du,

Sens. Actuators, B **2019**, *284*, 421.

[18] H. Tian, H. Fan, M. Li, L. Ma, *ACS Sensors* **2016**, *1*, 243.

[19] F. Qu, S. Zhang, C. Huang, X. Guo, Y. Zhu, T. Thomas, H. Guo, J. P. Attfield, M. Yang, *Angew. Chem. Int. Edit* **2021**, *60*, 6561.

[20] S. Luo, R. Chen, J. Wang, D. Xie, L. Xiang, *Sens. Actuators, B* **2021**, *344*, 130220.

[21] L. Lu, X. Wei, Y. Zhang, G. Zheng, K. Dai, C. Liu, C. Shen, *J. Mater. Chem. C* **2017**, *5*, 7035.

[22] X. Hu, F. Yang, M. Wu, Y. Sui, D. Guo, M. Li, Z. Kang, J. Sun, J. Liu, *Adv Mater Technol- US* **2022**, *7,* 2100769.

[23] J. Xu, H. Wang, T. Ma, Y. Wu, R. Xue, H. Cui, X. Wu, Y. Wang, X. Huang, W. Yao, *Carbon* **2020**, *166*, 316.

[24] U.-H. Shin, D.-W. Jeong, S.-M. Park, S.-H. Kim, H. W. Lee, J.-M. Kim, *Carbon* **2014**, *80*, 396.

[25] K. Chatterjee, J. Tabor, T. K. Ghosh, *Fibers* **2019**, *7*, 51.

[26] J. Shi, S. Lv, L. Wang, Z. Dai, S. Yang, L. Zhao, H. Tian, M. Du, H. Li, Y. Fang, *Adv. Mater. Interfaces* **2019**, *6*, 1901223.

[27] M. B. Azizkhani, J. Kadkhodapour, S. Rastgordani, A. P. Anaraki, B. S. Hadavand, *Fibers Polym* **2019**, *20*, 35.

[28] S. Zhu, H. Sun, Y. Lu, S. Wang, Y. Yue, X. Xu, C. Mei, H. Xiao, Q. Fu, J. Han, *ACS Appl. Mater. Interfaces* **2021**, *13,* 59142.

[29] M. Lei, K. Feng, S. Ding, M. Wang, Z. Dai, R. Liu, Y. Gao, Y. Zhou, Q. Xu, B. Zhou, *ACS Nano* **2022**, *16*, 12620.

[30] Y. Zheng, Y. Li, K. Dai, Y. Wang, G. Zheng, C. Liu, C. Shen, *Compos Sci Technol* **2018**, *156*, 276.
